# Voltage‐gated calcium channel activity of gonadotropin‐releasing hormone (GnRH) neurons is altered by age and by prenatal androgen exposure in female mice

**DOI:** 10.1111/jne.70268

**Published:** 2026-09-18

**Authors:** Xi Chen, Jennifer Jaime, R. Anthony DeFazio, Suzanne M. Moenter

**Affiliations:** ^1^ Department of Molecular & Integrative Physiology University of Michigan Ann Arbor Michigan USA; ^2^ The Neuroscience Graduate Program University of Michigan Ann Arbor Michigan USA; ^3^ Department of Internal Medicine University of Michigan Ann Arbor Michigan USA; ^4^ Obstetrics and Gynecology University of Michigan Ann Arbor Michigan USA; ^5^ Present address: Department of Pediatrics University of Colorado, Anschutz Medical Campus Aurora Colorado USA

**Keywords:** patch‐clamp, polycystic ovary syndrome, polyendocrine metabolic ovarian syndrome, puberty, reproduction

## Abstract

Polyendocrine metabolic ovarian syndrome (PMOS), a common cause of infertility, is marked by persistently high luteinizing hormone (LH) pulse frequency, presumably driven by high‐frequency GnRH pulses. Prenatally androgenized (PNA) mice mimic neuroendocrine PMOS symptoms including high LH‐pulse frequency. GnRH neurons from adult PNA mice have a higher spontaneous firing rate than those from vehicle (VEH) mice; this is reversed in peripubertal mice despite more excitatory inputs at both ages. We hypothesized voltage‐gated Ca^2+^ currents (*I*_Ca_) help set intrinsic excitability of GnRH neurons and are altered by development and/or PNA treatment. Whole‐cell patch‐clamp measurements were made of GnRH neuron *I*_Ca_ and excitability in 3 week‐old and adult VEH and PNA mice. PNA treatment increased *I*_Ca_ density and depolarized the *I*_Ca_‐half‐inactivation potential at both ages. In adults, the half‐activation potential of the Ca^2+^ conductance was depolarized, and the half‐inactivation time of the fast *I*_Ca_ component was increased regardless of PNA treatment. There was an age‐related change in firing response to current injections driven primarily by more firing in 3 week‐old VEH mice; 3 week‐old PNA mice had adult‐like responses. Blocking small‐conductance Ca^2+^‐activated K^+^ current with apamin increased GnRH neuron firing rate except in adult PNA mice. Apamin changed the post‐spike‐train membrane response from hyperpolarization to depolarization; during development, this net effect of apamin was smaller in PNA mice. In summary, GnRH neurons from PNA mice have increased *I*_Ca_ and altered *I*_Ca_ kinetics. Ca^2+^‐activated K^+^ currents are less prominent in GnRH neurons from adult PNA mice, perhaps contributing to their increased spontaneous firing activity.

## 1 INTRODUCTION

In vertebrates, pulsatile GnRH secretion from neurons in the preoptic area (POA) and hypothalamus is the ultimate neural mechanism that regulates reproduction. GnRH stimulates secretion of luteinizing hormone (LH) and follicle‐stimulating hormone (FSH) from the anterior pituitary^1^, ^2^, ^3^; LH and FSH control steroidogenesis and steroids feed back to regulate GnRH, LH and FSH release. GnRH‐pulse frequency varies during the female reproductive cycle and helps drive differential LH and FSH release. GnRH frequency is relatively low in the early follicular phase favoring FSH and higher in the late follicular phase favoring LH.^4^, ^5^, ^6^, ^7^ Disruption of GnRH/LH patterns can lead to infertility as in polyendocrine metabolic ovarian syndrome (PMOS), previously termed polycystic ovary syndrome (PCOS).^8^ PMOS is the most common endocrine fertility disorder in females. Hyperandrogenemic PMOS patients exhibit persistently high LH‐pulse frequency, presumably driven by high‐frequency GnRH pulses, and are resistant to sex steroid negative feedback.^9^, ^10^ The high LH‐pulse frequency exacerbates ovarian androgen production and can lead to relative FSH deficiency, impairing follicular maturation.^6^, ^11^ The hormone dysregulation may begin before or during the pubertal transition, as hyperandrogenemic adolescent girls exhibit higher LH‐pulse frequency, and half are resistant to progesterone inhibition.^11^, ^12^, ^13^, ^14^

The etiology of PMOS is not fully understood. Besides possible genetic causes, in utero androgen exposure could reprogram the fetus and produce PMOS‐like characteristics in adulthood.^15^ Consistent with this postulate, women with PMOS have higher androgen levels during late gestation^16^, ^17^, ^18^ and prenatal androgen (PNA) treatment of several species, including monkeys,^19^ sheep,^20^ rats^21^ and mice^22^, ^23^ results in female offspring with disrupted reproductive cycles, elevated testosterone levels, increased LH‐pulse frequency, and resistance to steroid negative feedback. Consistent with elevated LH‐pulse frequency,^24^ PNA mice exhibit increased spontaneous GnRH neuron action potential firing rate, but interestingly, only in adulthood. In contrast to control female mice, which exhibit a peak of GnRH neuron firing activity at 3 weeks of age before declining to adult levels, GnRH neuron firing rate is similar throughout development in PNA mice; as a result, firing rate is lower than controls at 3 weeks of age.^25^ These changes in firing rate likely represent integration of synaptic and intrinsic changes. In this regard, GABAergic fast synaptic transmission to GnRH neurons, which can be excitatory, is elevated at both 3 weeks of age and in adults.^22^, ^24^, ^26^, ^27^ GnRH neuron K^+^‐current properties change with both development and PNA treatment,^28^ whereas intrinsic excitability of GnRH neurons when intracellular Ca^2+^ is buffered is primarily altered by developmental stage, and not PNA treatment.^29^

Voltage‐gated Ca^2+^ currents play important roles in mediating excitation‐secretion coupling in GnRH neurons,^30^ and regulating GnRH neuron burst firing,^31^ which may influence GnRH pulse frequency and/or amplitude. It is unknown if Ca^2+^ currents are altered by development and/or PNA treatment. Here we investigated if there are changes in voltage‐gated Ca^2+^ currents in peripubertal versus adult GnRH neurons from control mice, and if PNA treatment alters any Ca^2+^ current properties. We also tested if the small‐conductance Ca^2+^‐activated K^+^ currents influence GnRH neuron excitability in control and PNA mice before and after puberty.

## 2 MATERIALS AND METHODS

All chemicals were acquired from Sigma‐Aldrich (St. Louis, MO, USA) unless noted otherwise.

### 2.1 Animals

Mice with green fluorescent protein (GFP) expression targeted to GnRH neurons (GnRH‐GFP mice, CBA‐Tg(Gnrh1‐EGFP)51Sumo/J, MGI:6158457; C57Bl6/J background) were bred in our colony (Suter et al., 2000). Mice were housed on a 14 h‐light 10 h‐dark photoperiod with lights on at 3 am (eastern standard time). Harlan 2916 (non‐breeders) or 2919 (breeders) chow and water were available ad libitum. To generate PNA mice, a homozygous GnRH‐GFP female was bred with a C57BL/6J male (Jackson Labs 000664). A companion CD1 female was included, bred, and gave birth. Companion dams provide maternal care and nutrition and increase survival of PNA pups. The first day of pregnancy in females was determined by the appearance of a copulatory plug during daily checks. On day 16 to day 18 of pregnancy, the GnRH‐GFP dam received 225 μg dihydrotestosterone (DHT) in 50 μL sesame oil sc for PNA treatment, or sesame oil vehicle (VEH) for controls. Mice were weaned at post‐natal day (PND) 21. CD1 pups, identified by coat color, were culled as needed to avoid crowding. All procedures were approved by the Institutional Animal Care and Use Committee of the University of Michigan.

### 2.2 Verification of PNA phenotype

The PNA status was confirmed by the age of vaginal opening (VO), the anogenital distance (AGD) measured at PND 70‐72, and estrous cyclicity via vaginal lavage monitored at PND 70‐83. For mice recorded on or before PND 21, the PNA status was confirmed by phenotypes of littermates raised to adulthood, as these phenotypes are consistent among littermates.

### 2.3 Brain slice preparation

Brain slices were prepared as described.^28^, ^32^ All bath solutions were bubbled with 95% O_2_/5% CO_2_ for at least 30 min before exposure to tissue and throughout experiments. The mouse brain was quickly removed and placed in the ice‐cold sucrose saline containing 250 mM sucrose (Invitrogen), 3.5 mM KCl (Fluka), 26 mM NaHCO_3_, 10 mM D‐glucose, 1.25 mM Na_2_HPO_4_·7H_2_O (Mallinkrodt), 1.2 mM MgSO_4_·7H_2_O (EMD Millipore), and 2.5 mM MgCl_2_ (Fluka), at pH 7.6 and 345 mOSM. Coronal brain slices (300 μm) through the POA of the hypothalamus were cut using a VT1200 S vibratome (Leica Biosystems). Brain slices were kept in a 1:1 mixture of sucrose saline and artificial cerebrospinal fluid (ACSF) containing 135 mM NaCl (Fluka), 3.5 mM KCl, 26 mM NaHCO_3_, 1.25 mM Na_2_HPO_4_·7H_2_O, 2.5 mM CaCl_2_, 1.2 mM MgSO_4_·7H_2_O, and 10 mM D‐glucose at pH 7.4 and 305 mOSM, at room temperature for 30 min. Brain slices were then transferred to 100% ACSF and incubated for 0.5–6 h before recording. No differences were noted in data based upon time after slice preparation within this range.

### 2.4 Recording solutions and data acquisition

During recording, the brain slice was placed in a chamber and consistently perfused (1.5‐2 mL/min) with solutions (see below) kept between 29 and 31°C with an inline heater (Warner Instruments, Hamden, CT). GFP‐positive GnRH neurons were identified by a brief illumination at 470 nm using an Olympus BX51WI microscope. Whole‐cell voltage‐clamp and current‐clamp recordings were made using one channel of a HEKA dual EPC10 amplifier running PatchMaster (HEKA Elektronik). Data were sampled at 10 kHz and filtered at 7.3 kHz.

For recording Ca^2+^ currents, brain slices were perfused with a solution containing: 120 mM NaCl, 10 mM D‐glucose, 26 mM NaHCO_3_, 1.25 mM Na_2_HPO_4_, 1.2 mM MgSO_4_, 1.0 mM CaCl_2_, 1.5 mM MgCl_2_, 5 mM 4‐aminopyridine (4‐AP), 5 mM CsCl, 10 mM TEA‐Cl, 2 μM TTX (Tocris), pH 7.7 and 305 mOSM. Pulled capillary glass pipette (3–3.9 MΩ) was filled with a solution containing 120 mM Cs‐gluconate, 10 mM HEPES, 10 mM EGTA, 0.5 mM CaCl_2_, 4 mM Mg‐ATP, 0.4 mM Na‐GTP, 20 mM TEA‐Cl, pH 7.2 and 301 mOSM. The pipette was placed at the surface of a GnRH neuron soma and a seal of >1 GΩ was formed with the cell membrane. After stabilizing for 3 min, a gentle suction was applied to achieve the whole‐cell configuration. Cells were stabilized in the whole‐cell configuration for 30 s to allow dialysis of the pipette solution and were held at −70 mV between protocols. Slow capacitive current was electronically compensated, and *R*_s_ was auto‐compensated with prediction of 50%–75%. Recordings were corrected online for a liquid junctional potential of −13.3 mV. Leak current was subtracted online (*P*/−10) using an average of 6 sweeps from a baseline potential of −70 mV.^33^ Before and after a series of voltage steps, input resistance (*R*_in_), series resistance (*R*_s_), holding current (*I*_hold_) and capacitance (C_m_) were measured in voltage‐clamp mode from the mean current response to 20 5 mV‐hyperpolarizing steps from −70 mV. Cells had high *R*_in_ attributable to the blockage of potassium channels. Recordings were analyzed only if the following quality parameters were met: C_m_ 8 to 20 pF, *R*_in_ >900 MΩ, stable *R*_s_< 22 MΩ, *I*_hold_ of −55 to 0 pA. Pilot studies were conducted to examine rundown of peak current and based on these all recordings were completed within 4 min of achieving the whole‐cell configuration.

Action potentials during current injections were recorded to characterize excitability. To evaluate the excitability of GnRH neurons without extrinsic calcium buffering, pipettes were filled with EGTA‐free solution containing 136.5 mM K gluconate, 20.4 mM NaCl, 10.5 mM HEPES, 4.2 mM MgATP, 0.4 mM NaGTP, 10.3 mM KOH, pH 7.2 with NaOH, 310 mOSM. Brain slices were perfused with ACSF containing 20 μM D‐APV (Tocris), 10 μM CNQX (Tocris), and 100 μM picrotoxin to block ionotropic glutamate and GABA receptors. After achieving the whole‐cell configuration as described above, series resistance was calculated during slow‐capacitance compensation for an estimation of bridge balance, and the recording mode was transferred to current‐clamp with a bridge balance of 95%. Additional direct current (<5 pA) was injected if needed to keep the baseline membrane potential near −70.7 ± 0.1 mV (range: −72.9 to −68.4 mV). Recordings were corrected online for a liquid junctional potential of −14.4 mV and were analyzed only if the following quality parameters were met: C_m_ 8 to 20 pF, *R*_in_ >500 MΩ, stable *R*_s_< 25 MΩ, *I*_hold_ of −30 to 10 pA.

### 2.5 Experimental design

Calcium currents and excitability of GnRH neurons were measured in peripubertal mice (PND 18–21 before weaning, referred to as 3 week) and adult mice (PND 84‐155). Mice were treated prenatally with either VEH or DHT resulting in four groups: 3 week VEH, 3 week PNA, adult VEH and adult PNA. Adult females were recorded on diestrus determined by vaginal cytology and confirmed by uterine mass. Diestrus was chosen as PNA mice do not exhibit regular cycles; rather, they have cytology most representative of diestrus most of the time. For each group, a minimum of five mice from at least four litters were used. No more than three GnRH neurons from the same mouse were analyzed.

### 2.6 Voltage‐gated Ca^2+^ current characterization

Ca^2+^ currents were isolated pharmacologically by using nominally K^+^‐free pipette and external solutions and blocking voltage‐gated K^+^ (4‐AP and TEA‐Cl) and Na^+^ (TTX) channels. Pilot studies indicated that depolarization at −40 mV for 200 ms inactivates the fast‐inactivating component of the Ca^2+^ current, and hyperpolarization at −100 mV for 2000 ms completely removes inactivation. Fast and medium‐slow‐inactivating components of Ca^2+^ current (fast *I*_Ca_ and medium‐slow *I*_Ca_, respectively) were thus distinguished based on voltage dependence and time course.

### 2.7 Voltage‐dependent activation of Ca^2+^ currents

To measure total Ca^2+^ current, the membrane potential was hyperpolarized to −100 mV for 2000 ms to remove inactivation, followed by an additional 200 ms prepulse at −100 mV. The cell was then given a series of 200 ms voltage steps from −100 mV to +60 mV in 10 mV increments. After this series, the same cell received the same voltage step protocol, but with the 200 ms prepulse at −40 mV (rather than −100 mV) to inactivate the fast *I*_Ca_ to reveal the medium‐slow *I*_Ca_ components. The fast *I*_Ca_ was mathematically isolated by subtracting the medium‐slow *I*_Ca_ obtained with the −40 mV prepulse from total current obtained with the −100 mV prepulse.

### 2.8 Voltage‐dependent inactivation of fast *I*_Ca_

Depolarization to 0 mV produced the largest macroscopic Ca^2+^ current in GnRH neurons and was thus used as the test pulse to evaluate voltage‐dependent inactivation of fast *I*_Ca_. After hyperpolarization to −100 mV to remove inactivation, the GnRH neuron was subjected to a 200 ms prepulse from −90 mV to −40 mV at 5 mV or 10 mV increments, followed by a test pulse at 0 mV for 25 ms; data from the two increments were consistent and combined. The more depolarized prepulses led to a smaller current triggered by the 0 mV test pulse, reflecting voltage‐dependent inactivation of fast *I*_Ca_. The fast *I*_Ca_ was isolated by subtracting the test current at 0 mV following the −40 mV prepulse from test currents with prepulses of −90 to −45 mV.

### 2.9 Time‐dependent inactivation and recovery of fast *I*_Ca_

To measure the time course of inactivation of the fast *I*_Ca_, GnRH neurons were hyperpolarized to −100 mV for 2000 ms to remove inactivation and then depolarized to the inactivation potential of −40 mV for 1, 2, 4, 8, 16, 32, 64, 128, and 256 ms, followed by a test pulse at 0 mV for 25 ms. The non‐inactivating component during the test pulse after the 256 ms inactivation was subtracted from currents of other sweeps with shorter inactivation times to isolate the fast *I*_Ca_. To measure the time course of recovery from inactivation of fast *I*_Ca_, GnRH neurons were first depolarized to −40 mV for 200 ms to inactive this component. The cell was then subjected to −100 mV hyperpolarization for 0, 1, 2, 4, 8, 16, 32, 64, 128, 256, 512, 1024, and 2048 ms to recover the fast *I*_Ca_, followed by a test pulse at 0 mV for 25 ms. The current during the test pulse without a recovery prepulse (0 ms) was subtracted from currents of other sweeps with longer time of recovery to isolate the fast *I*_Ca_.

### 2.10 Intrinsic excitability of GnRH neuron

To evaluate GnRH neuron intrinsic excitability, the firing response to sequential current injections (0–100 pA, 10 pA increment, 500 ms) was recorded using whole‐cell current‐clamp without EGTA buffering of the pipette solution so that the potential influence of Ca^2+^ influx and/or release from intracellular stores was not hindered; additional cells were recorded with EGTA buffering for pilot comparisons. Under EGTA‐free conditions, GnRH neurons presented a prolonged, slow hyperpolarization following the termination of current injection, which gradually returned to baseline potential over several seconds. The interval between current steps was thus set to 15 s so the membrane potential would return to approximately −70 mV before the next step was initiated.

### 2.11 The effect of small conductance Ca^2+^‐activated K^+^ (SK) current on GnRH neuron excitability

Apamin (300 nM, Tocris) was applied to block the small‐conductance Ca^2+^‐activated K^+^ channels to investigate the role of these channels in GnRH neuron excitability. Pilot studies determined the response to apamin continued to develop over 6 min and was most evident at 80 pA; longer recordings were precluded by current rundown. To test the effect of apamin, action potentials were first recorded under basal state (0 min) in response to an 80 pA 500 ms‐current injection. Then, the brain slice was perfused with apamin or ACSF control and the response to identical current injection was recorded 6 min later. The difference in spike number was calculated between 6 min and pretreatment. Passive properties of the GnRH neuron were recorded in response to the average of 20 5 mV hyperpolarizing steps from −70 mV before and after each train of current injections to verify recording stability. UCL 2077 (10 μM, Tocris), a blocker of KCNQ1 and KCNQ2 potassium channels,^31^, ^34^ was tested in pilot studies but had no discernable effect on action potential firing under these conditions (data not shown).

### 2.12 Analysis

For Ca^2+^ current analysis, the current at each voltage step was normalized by cell capacitance to yield current density. To make *I*–*V* curves, current densities of total *I*_Ca_, medium‐slow *I*_Ca_, and fast *I*_Ca_ were plotted against each command voltage. Because of the potential influence of unblocked potassium currents from 30 to 60 mV, statistical analysis for current density and voltage‐dependent activation was limited to the range of −100 to 20 mV.

To assess the voltage‐dependent activation of total, fast, and medium‐slow Ca^2+^ current, Ca^2+^ conductance was calculated by dividing the current peak of each voltage step by the driving force, which is derived from the Goldman–Hodgkin–Katz (GHK) equation:

$$\mathrm{GHK}\left(V\right)=V\left(\frac{\mathrm{zF}}{\mathrm{RT}}\right)\frac{\left(\exp\left(\frac{\mathrm{zF}}{\mathrm{RT}}\left(V-E_{\mathrm{Ca}}\right)\right)-1\right)}{\left(\exp\left(\frac{\mathrm{zF}}{\mathrm{RT}}V\right)-1\right)}$$

where *V* is the step potential, *R* is the universal gas constant, *T* is the temperature in Kelvin, *z* is the valence of Ca^2+^, *F* is the Faraday's constant, and *E*_Ca_ was calculated to be 151.43 mV.

The Ca^2+^ conductance was normalized to the maximum value and plotted as a function of potential to reveal voltage‐dependence of activation. The activation curve was fitted with the Boltzmann equation:

$$I\left(V\right)=\frac{1}{1+\exp\left(\frac{V_{0.5}-V}{k}\right)}$$

where *V*_0.5_ is the potential that the conductance reaches the half maximum (*V*_0.5 act_), and *k* is the slope factor. *V*_0.5 act_ and *k* were used to assess the activation of Ca^2+^ current.

To assess the voltage‐dependent inactivation of fast *I*_Ca_, the peak of fast *I*_Ca_ obtained at 0 mV with varying prepulses was normalized to maximum fast *I*_Ca_, defined as the average of currents following −80, −75, and −70 mV prepulses, as voltage‐dependent inactivation did not occur in this range. The normalized fast *I*_Ca_ was plotted against the corresponding command voltage step and fitted to the Boltzmann equation as above; *V*_0.5 inact_ and *k* were used to assess the inactivation of fast *I*_Ca_.

To assess the time‐dependent inactivation of fast *I*_Ca_, the peak fast *I*_Ca_ after 1–256 ms inactivation prepulses was normalized to the maximum value after 1 ms of inactivation, and plotted as a function of inactivation time. The time‐dependent inactivation curve was fitted to the monophasic decay equation:

$$Y=\exp\left(-\mathrm{kx}\right)$$

where *k* is the rate constant. The time at which half of the fast *I*_Ca_ was inactivated by −40 mV depolarization (*T*_0.5 inact_) is computed as ln(2)/*k*.

To assess the time‐dependent recovery of fast *I*_Ca_ after inactivation, the peak fast *I*_Ca_ after 1–2048 ms recovery prepulses was normalized to the maximum value after 2048 ms of recovery and plotted as a function of recovery time. The time‐dependent recovery curve was fitted to the monophasic association equation:

$$Y=1-\exp\left(-\mathrm{kx}\right)$$

where *k* is the rate constant and *T*_0.5 recov_ was computed as ln(2)/*k*.

To assess GnRH neuron excitability, firing rate was plotted as a function of current injection amplitude. The first spike fired at rheobase was used to profile action potential characteristics. Action potential latency from the start of current injection, threshold (membrane potential at which the second derivative is >5000 mV/s/s), rate of rise, peak amplitude relative to threshold, full width at half‐maximum (FWHM), time and amplitude of the afterhyperpolarization potential (AHP) relative to threshold were compared among groups.

Under basal conditions, all GnRH neurons exhibited hyperpolarization after a train of action potentials triggered by the current injection. To analyze the fast and slow post‐spike‐train membrane potential change relative to the pre‐stimulus baseline potential (Δ*V*_m_), the point at which the repolarization rate after current injection termination fell below 50 mV/s was used; the period before being designated fast Δ*V*_m_ and that after slow Δ*V*_m_. Peak values of fast and slow Δ*V*_m_ were compared among groups. The area under the curve (AUC) of Δ*V*_m_ during the post‐stimulus 12.5 s period were compared.

To compare the post‐spike‐train membrane depolarization that emerged with 6 min apamin treatment, the post‐spike‐train depolarization peak and the latency to the peak following termination of current injection were compared. AUC and duration of the post‐spike‐train depolarization were quantified. To minimize the influence of noise and prolonged tails, the onset and termination were defined as the points when potential crossed 10% of the depolarization peak value (AUC at 10% maximum, duration at 10% maximum).

To assess the net effect of apamin‐sensitive SK current on post‐spike‐train membrane potential, the membrane potential trace recorded starting 6 min into apamin treatment was subtracted from that recorded before treatment in the same cell. The SK‐mediated hyperpolarization peak value, peak latency, AUC at 10% maximum, and duration at 10% maximum were analyzed.

### 2.13 Statistics

Statistics were performed by Igor 9 (Sutter, WaveMetrics), Prism 9 (GraphPad LLC.) and R Studio (LMER test, LME4 and emmeans packages implemented in version 4.4.0). Data were tested for normality using the Shapiro–Wilk test. Equality of variance was tested by F test. Data were reported as the mean ± SEM (normal distribution) or median ± IQR (non‐normal distribution). Details of numbers of cells and animals, and of statistical tests are provided in the results and tables.

## 3 RESULTS

### 3.1 Verification of prenatal androgenization phenotype

**FIGURE 1:**
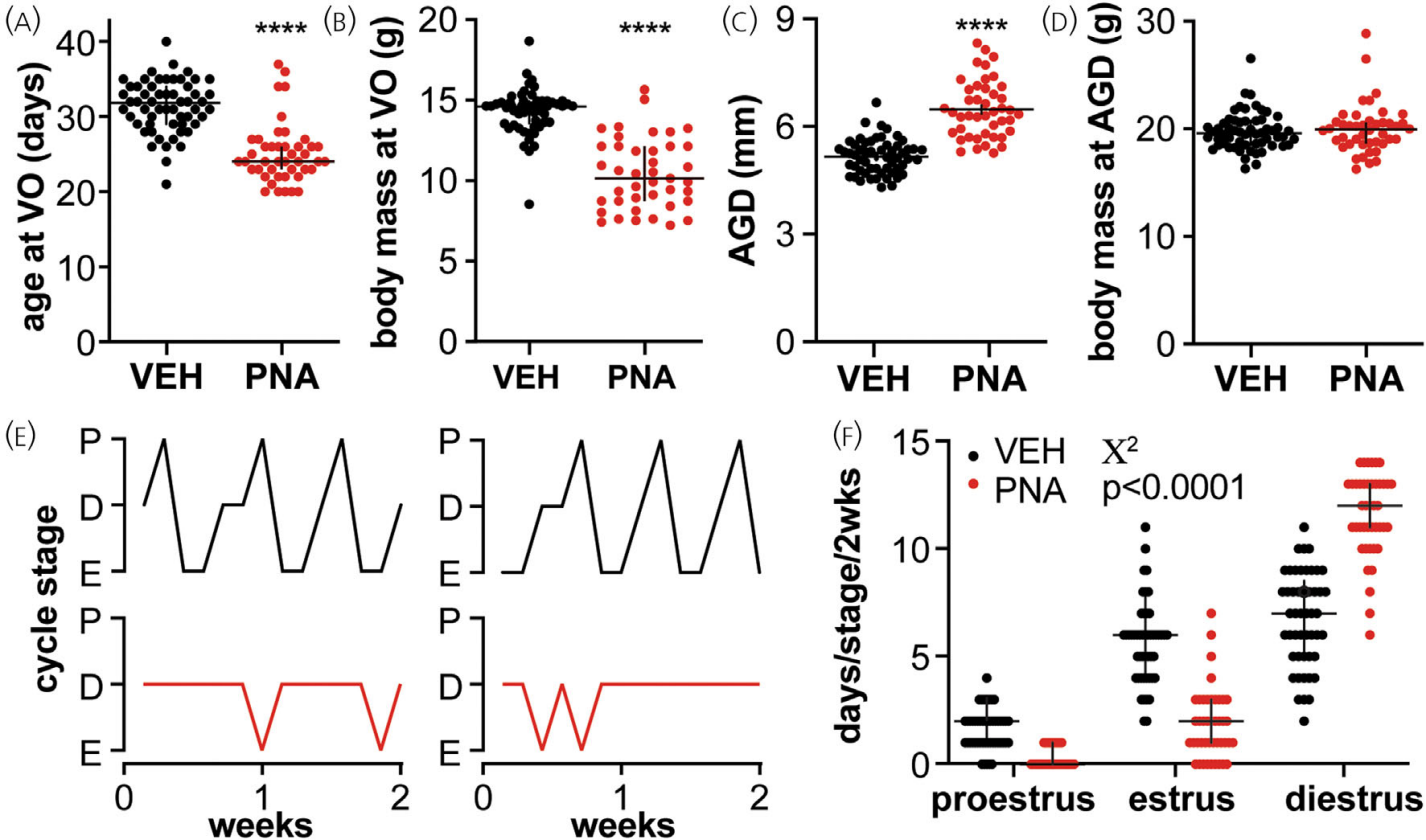
Verification of PNA phenotype. (A), (B), (D), Individual values and median ± interquartile range for age at vaginal opening (VO) (A), body mass at the day of VO (B), and body mass at PND 70‐72 (D). (C) Individual values and mean ± SEM for anogenital distance (AGD) measured at postnatal day (PND) 70‐72. (E) Representative estrous cycles of two VEH (top, black) and two PNA (bottom, red) mice over 14 days. P, proestrus; D, diestrus; E, estrus. Days in proestrus, estrus and diestrus stages over 14 days. ****p< .0001, two‐tailed Mann–Whitney *U* test (A, B, D), two‐tailed unpaired Welch's *t*‐test (C), Χ^2^ (F).

PNA‐induced differences were confirmed by the age of VO, AGD, and estrous cyclicity. Consistent with previous observations,^22^, ^25^, ^28^, ^29^ PNA mice exhibited earlier VO (Figure 1A, two‐tailed Mann–Whitney *U* test, *U* = 265.6, *p*< .0001), which occurred at a lower body mass (Figure 1B, two‐tailed Mann–Whitney *U* test, *U* = 157, *p*< .0001). At PND70‐72, PNA mice had increased anogenital distance (AGD, Figure 1C, two‐tailed unpaired Welch's *t*‐test, *t* = 10.19, df = 98, *p*< .0001), while body mass did not differ from VEH (Figure 1D, two‐tailed Mann–Whitney *U* test, *U* = 1171). Adult PNA mice had disrupted estrous cycles, with more days in diestrus and fewer days in proestrus and estrus compared with controls (Figure 1E,F, Chi‐square test *p*< .0001, *Χ* ^2^ = 190.8, df = 2). While PNA mice undergo earlier vaginal opening, perhaps reflecting earlier age at menarche in women with PMOS,^35^ the majority of 3 week old mice had not undergone vaginal opening at the time of study. Comparison of the same ages in VEH and PNA mice may mean PNA mice are closer to vaginal opening, but there are no external markers that can provide this information and no way to know how many days would pass before VO if mice had not been used in experiments.

### 3.2 Ca^2+^ current density in GnRH neurons is increased by PNA treatment

Ca^2+^ currents were recorded using whole‐cell voltage‐clamp. Passive properties and series resistance of voltage‐clamp recordings were used to assess the recording quality and cell properties. Because potassium channels were blocked, all cells exhibited high input resistance (*R*_in_ >900 MΩ). The cell capacitance was higher in the adult groups compared to the 3 week‐old groups, but PNA treatment had no effect (Table 1). There were no differences in *R*_in_, *R*_s_, or *I*_hold_ among groups.

**TABLE 1:** Descriptive statistics and two‐way ANOVA parameters for passive properties and recording quality for Ca^2+^ currents recording (Figures 2, 3, 4).

Descriptive statistics				
Property	3 week VEH	3 week PNA	Adult VEH	Adult PNA
Series resistance (MΩ)	14.869 ± 0.481	14.642 ± 0.465	15.810 ± 0.466	15.402 ± 0.490
Input resistance (MΩ)	1634.412 ± 78.424	1545.381 ± 73.494	1684.143 ± 84.791	1530.916 ± 61.880
Capacitance (pF)	12.943 ± 0.444	13.882 ± 0.405	16.258 ± 0.480	16.181 ± 0.484
Holding current (pA)	−31.598 ± 1.471	−32.065 ± 1.537	−28.319 ± 1.381	−30.591 ± 1.913

Two‐way ANOVA			
	Age	Treatment	Interaction
Series resistance (MΩ)	*F* (1, 119) = 3.193, *p* = .0765	*F* (1, 119) = 0.4455, *p* = .5058	*F* (1, 119) = 0.03638, *p* = .8491
Input resistance (MΩ)	*F* (1, 119) = 0.05504, *p* = .8149	*F*(1, 119) = 2.597, *p* = .1097	*F* (1, 119) = 0.1824, *p* = .6701
Capacitance (pF)	***F* (1, 119) = 38.03**, ***p*< .0001**	*F* (1, 119) = 0.8962, *p* = .3457	*F* (1, 119) = 1.224, *p* = .2669
Holding current (pA)	*F* (1, 119) = 2.229, *p* = .1381	*F* (1, 119) = 0.7461, *p* = .3894	*F* (1, 119) = 0.5700, *p* = .3244

Holm‐Sidak analysis of significant two‐way ANOVA				
	3 week VEH versus 3 week PNA	Adult VEH versus adult PNA	3 week VEH versus adult VEH	3 week PNA versus adult PNA
Capacitance (pF)	*p* = .2759	*p* = .9047	***p*< .0001**	***p* = .0016**

*Note*: Bold indicates *p*< .05.

**FIGURE 2:**
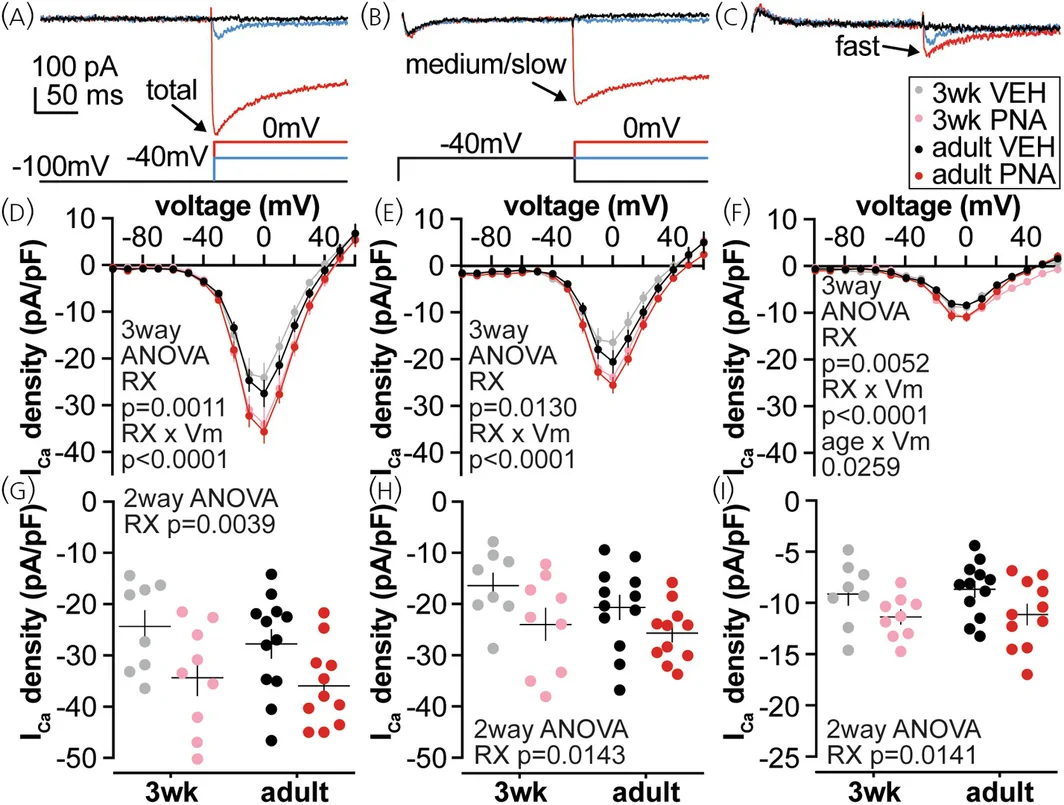
Ca^2+^ currents of GnRH neurons from VEH and PNA mice during development. (A)–(C), Representative current traces (above) and voltage commands (below) for total *I*_Ca_ (A), medium‐slow *I*_Ca_ (B), and mathematically‐isolated fast *I*_Ca_ (C) from an adult PNA mouse. Labeled arrows (total, medium‐slow, fast) identify current components. The 2000 ms prepulse at −100 mV to remove inactivation is not shown. Only three voltage steps (−100, −40, 0 mV) from the voltage family from −100 to 60 mV are shown for clarity. (D)–(F), Current density–voltage (*I*–*V*) curve for total *I*_Ca_ (D), medium‐slow *I*_Ca_ (E), and mathematically‐isolated fast *I*_Ca_ (F). Note the *Y*‐axis does not cross the *X*‐axis at zero. (G)–(I), current densities of total *I*_Ca_ (G), medium‐slow *I*_Ca_ (H), and mathematically‐isolated fast *I*_Ca_ (I) at 0 mV. RX, prenatal treatment.

The total *I*_Ca_ in GnRH neurons is comprised of two main components: a fast‐inactivating component (fast *I*_Ca_) that was inactivated by a 200 ms prepulse at −40 mV, and a medium‐slow inactivating component (medium‐slow *I*_Ca_) that remains after the −40 mV inactivation. Representative traces of total *I*_Ca_, medium‐slow *I*_Ca_ and mathematically‐isolated fast *I*_Ca_ are shown in Figure 2A–C, current density–voltage (*I*–*V*) curves in Figure 2D–F and current densities at 0 mV in Figure 2G–I. PNA treatment altered the *I*–*V* curves of the total *I*_Ca_ and both individual components (Table 2). At 0 mV, where the maximum total *I*_Ca_ was observed, GnRH neurons from PNA mice exhibited higher total *I*_Ca_ density compared to VEH groups regardless of age. (Figure 2G, Table 3). Total *I*_Ca_ density at −20, −10, 10, and 20 mV was also greater in PNA groups (two‐way ANOVA PNA treatment: −20 mV *F* (1, 35) = 5.542, *p* = .0243; −10 mV *F* (1, 35) = 6.927, *p* = .0125; 10 mV *F* (1, 35) = 9.193, *p* = .0046; 20 mV *F* (1, 35) = 11.49, *p* = .0017). The greater total *I*_Ca_ was attributable to higher *I*_Ca_ densities of both medium‐slow and fast components in cells from PNA mice (Figure 2H,I, Table 3). Although three‐way ANOVA showed the *I*–*V* curve of the fast component was affected by age (age × voltage *p* = .0259), the main age‐dependent differences were observed from +30 to +60 mV. At this voltage range, unblocked potassium currents may influence results. This statistical difference is thus likely not biologically relevant and is not considered further.

**TABLE 2:** Three‐way ANOVA of current density–voltage (*I*–*V*) curve for total *I*_Ca_, medium‐slow *I*_Ca_, and mathematically‐isolated fast *I*_Ca_ (Figure 2).

Source of variation	Total *I*_Ca_	Medium‐slow *I*_Ca_	Fast *I*_Ca_
Voltage	***F* (16, 576) = 317.6**, ***p*< .0001**	***F* (16, 576) = 215.4**, ***p*< .0001**	***F* (16, 576) = 196.5**, ***p*< .0001**
Age	*F* (1, 36) = 0.6734, *p* = .4173	*F* (1, 36) = 1.450, *p* = .2363	*F* (1, 36) = 1.217, *p* = .2772
Treatment	***F* (1, 36) = 12.50**, ***p* = .0011**	***F* (1, 36) = 6.838**, ***p* = .0130**	***F* (1, 36) = 8.875**, ***p* = .0052**
Voltage × age	*F* (16, 576) = 0.6167, *p* = .8717	*F* (16, 576) = 1.219, *p* = .2475	***F* (16, 576) = 1.818**, ***p* = .0259**
Voltage × treatment	***F* (16, 576) = 6.883**, ***p*< .0001**	***F* (16, 576) = 4.335**, ***p*< .0001**	***F* (16, 576) = 4.010**, ***p*< .0001**
Age × treatment	*F* (1, 36) = 0.08796, *p* = .7685	*F* (1, 36) = 0.04491, *p* = .8334	*F* (1, 36) = 0.1523, *p* = .6986
Voltage × age × treatment	*F* (16, 576) = 0.2674, *p* = .9982	*F* (16, 576) = 0.4529, *p* = .9673	***F* (16, 576) = 1.756**, ***p* = .0338**

*Note*: Bold indicates *p*< .05.

**TABLE 3:** Two‐way ANOVA of current density at 0 mV (Figure 2).

Current type	Age	Treatment	Interaction
Total *I*_Ca_	*F* (1, 36) = 0.7165, *p* = .4029	***F* (1, 36) = 9.540**, ***p* = .0039**	*F* (1, 36) = 0.09465, *p* = .7601
Medium‐slow *I*_Ca_	*F* (1, 36) = 1.475, *p* = .2324	***F* (1, 36) = 6.625**, ***p* = .0143**	*F* (1, 36) = 0.2647, *p* = .6100
Fast *I*_Ca_	*F* (1, 36) = 0.1373, *p* = .7131	***F* (1, 36) = 6.653**, ***p* = .0141**	*F* (1, 36) = 0.01243, *p* = .9119

Holm‐Sidak analysis of significant two‐way ANOVA				
	3 week VEH versus 3 week PNA	Adult VEH versus adult PNA	3 week VEH versus adult VEH	3 week PNA versus adult PNA
Total *I*_Ca_	*p* = .1200	*p* = .1497	*p* = .8894	*p* = .9922
Medium‐slow *I*_Ca_	*p* = .1827	*p* = .3250	*p* = .4115	*p* = .6208
Fast *I*_Ca_	*p* = .3010	*p* = .1724	*p* = .9309	*p* = .9309

*Note*: Bold indicates *p*< .05.

### 3.3 Voltage‐dependent activation of the Ca^2+^ conductance in GnRH neurons is affected by development

**FIGURE 3:**
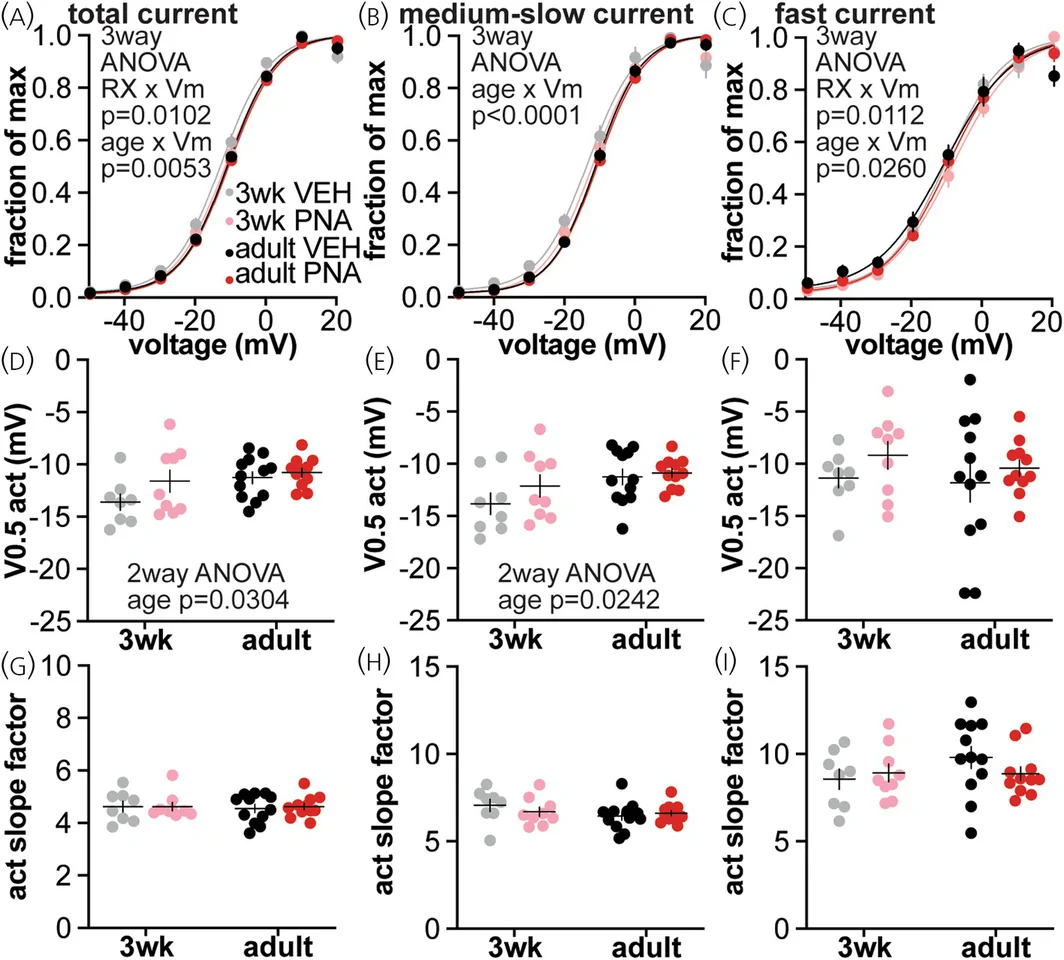
Voltage‐dependent activation of Ca^2+^ conductances of GnRH neurons from VEH and PNA mice during development. (A)–(C), voltage‐dependent activation of total (A), medium‐slow (B), and fast (C) Ca^2+^ conductance. Dots/error: Mean ± SEM of Ca^2+^ conductance normalized to the maximum value. Lines: Boltzmann fits of the voltage‐dependent activation curves of Ca^2+^ conductance. (D)–(F), half‐activation potential (*V*_0.5 act_) of total (D), medium‐slow (E), and fast (F) Ca^2+^ conductance. (G)–(I), activation slope factor of total (G), medium‐slow (H), and fast (I) Ca^2+^ conductance. RX, prenatal treatment.

Ca^2+^ conductance was calculated by dividing the peak current at each voltage step by the driving force derived from the GHK equation to assess voltage‐dependent activation. The Ca^2+^ conductance at each voltage step was normalized to the maximum current value, typically achieved at 10 or 20 mV, and plotted as a function of test potential (Figure 3A–C). Voltage interacted either with both age and treatment (total and fast conductance) or age (medium‐slow conductance, Figure 3A–C, Table 4). The potential at which the total and medium‐slow Ca^2+^ conductances reached the half maximum (*V*_0.5 act_) was depolarized in cells from adult mice compared with those from 3 week mice (Figure 3D,E, Table 5), whereas the *V*_0.5 act_ of fast Ca^2+^ was unchanged among groups (Figure 3F). The activation slope factor (*k*) was not influenced by either development or PNA treatment for any aspect of the calcium conductance (Figure 3G–I).

**TABLE 4:** Three‐way ANOVA test of voltage‐dependent activation of calcium conductances (Figure 3).

Source of variation	Total *I*_Ca_	Medium‐slow *I*_Ca_	Fast *I*_Ca_
Voltage	***F* (9, 333) = 4282**, ***p*< .0001**	***F* (9, 324) = 2818**, ***p*< .0001**	***F* (9, 324) = 1201**, ***p*< .0001**
Age	*F* (1, 37) = 3.601, *p* = .0656	*F* (1, 36) = 3.528, *p* = .0685	*F* (1, 36) = 0.3421, *p* = .5623
Treatment	*F* (1, 37) = 2.547, *p* = .1190	*F* (1, 36) = 2.002, *p* = .1657	*F* (1, 36) = 1.908, *p* = .1757
Voltage × age	***F* (9, 333) = 2.669**, ***p* = .0053**	***F* (9, 324) = 6.037**, ***p*< .0001**	***F* (9, 324) = 2.140**, ***p* = .0260**
Voltage × treatment	***F* (9, 333) = 2.454**, ***p* = .0102**	*F* (9, 324) = 1.245, *p* = .2666	***F* (9, 324) = 2.425**, ***p* = .0112**
Age × treatment	*F* (1, 37) = 0.5269, *p* = .4725	*F* (1, 36) = 1.124, *p* = .2962	*F* (1, 36) = 0.01069, *p* = .9812
Voltage × age × treatment	*F* (9, 333) = 0.4289, *p* = .9192	*F* (9, 324) = 0.2627, *p* = .9839	*F* (9, 324) = 0.7071, *p* = .7019

*Note*: Bold indicates *p*<.05.

**TABLE 5:** Two‐way ANOVA of activation properties (Figure 3).

V0.5 activation			
Current type	Age	Treatment	Interaction
Total *I*_*C*a_	***F* (1, 36) = 5.081**, ***p* = .0304**	*F* (1, 36) = 3.155, *p* = .0842	*F* (1, 36) = 1.123, *p* = .2964
Medium‐slow *I*_Ca_	***F* (1, 36) = 5.539**, ***p* = .0242**	*F* (1, 36) = 1.624, *p* = .2106	*F* (1, 36) = 0.6619, *p* = .4212
Fast *I*_Ca_	*F* (1, 36) = 0.3590, *p* = .5528	*F* (1, 36) = 1.595, *p* = .2147	*F* (1, 36) = 0.07312, *p* = .7884

Holm‐Sidak analysis of significant two‐way ANOVA				
	3 week VEH versus 3 week PNA	Adult VEH versus adult PNA	3 week VEH versus adult VEH	3 week PNA versus adult PNA
Total *I*_Ca_	*p* = .1950	*p* = .6403	*p* = .0993	*p* = .6403
Medium‐slow *I*_Ca_	*p* = .4430	*p* = .7256	*p* = .1242	*p* = .4813

Slope factor			
Current type	Age	Treatment	Interaction
Total *I*_Ca_	*F* (1, 36) = 0.05201, *p* = .8209	*F* (1, 36) = 0.04075, *p* = .8412	*F* (1, 36) = 0.04636, *p* = .8307
Medium‐slow *I*_Ca_	*F* (1, 36) = 1.981, *p* = .1678	*F* (1, 36) = 0.1477, *p* = .7030	*F* (1, 36) = 1.147, *p* = .2913
Fast *I*_Ca_	*F* (1, 36) = 1.190, *p* = .2826	*F* (1, 36) = 0.2757, *p* = .6028	*F* (1, 36) = 1.398, *p* = .2448

*Note*: Bold indicates *p*< .05.

### 3.4 Voltage‐ and time‐dependent inactivation, and time‐dependent recovery of fast *I*_Ca_ of GnRH neurons

**FIGURE 4:**
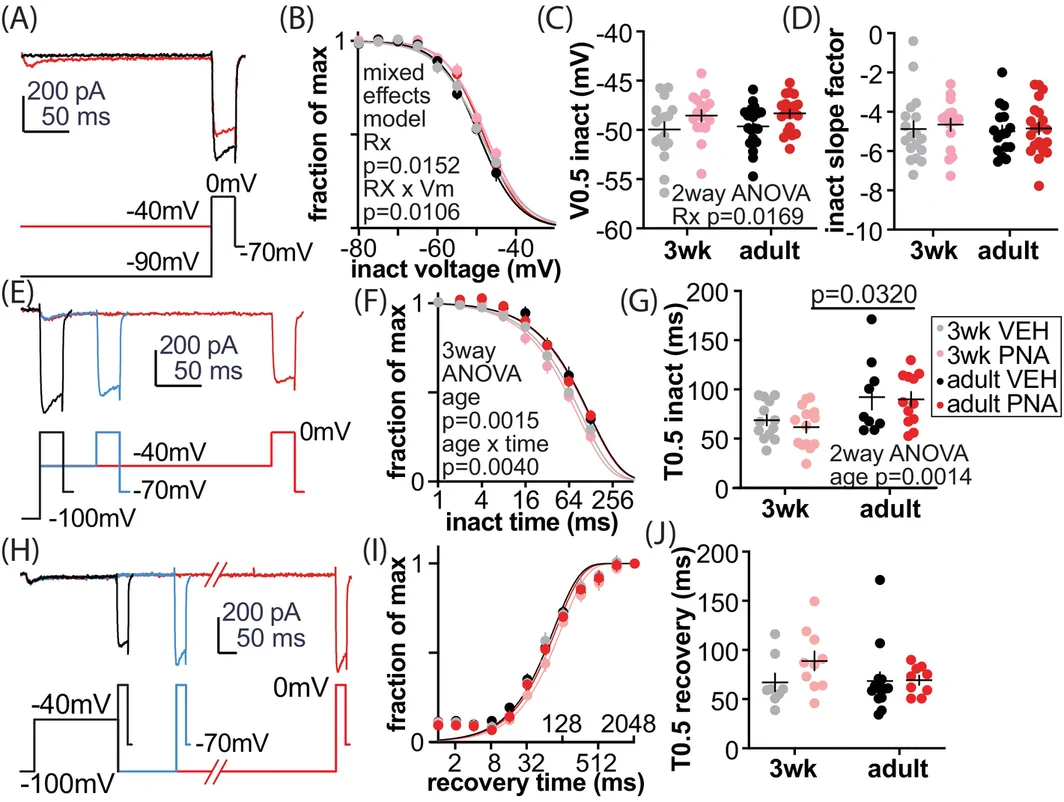
Voltage‐ and time‐dependent inactivation, and time‐dependent recovery of GnRH neuron fast *I*_Ca_ in VEH and PNA mice. (A) Representative current traces (above) and voltage commands (below) for voltage‐dependent inactivation of fast *I*_Ca_. Only two prepulses (−90 and −40 mV) are shown for clarity. (B) Dots/error: Mean ± SEM of fast *I*_Ca_ tested at 0 mV with different inactivation voltage prepulses. Data were normalized to the maximum value, which is the average of fast *I*_Ca_ with prepulses of −80, −75, and −70 mV. Lines: Boltzmann fits of the voltage‐dependent inactivation curves of fast *I*_Ca_. (C) Half‐inactivation potential (*V*_0.5 inact_) of fast *I*_Ca_. (D) Inactivation slope factor (*k*) of fast *I*_Ca_. (E) Representative current traces (above) and voltage commands (below) for time‐dependent inactivation of fast *I*_Ca_. Only three inactivation times (1, 64, 256 ms) are shown for clarity. (F) Dots/error: Mean ± SEM of fast *I*_Ca_ tested at 0 mV with different inactivation time on a log scale. Data were normalized to the maximum value obtained with 1 ms inactivation time. Lines: Monophasic exponential decay fits of time‐dependent inactivation curve. (G) Half‐inactivation time (*T*_0.5 inact_) of fast *I*_Ca_. (H) Representative current traces (above) and voltage commands (below) for time‐dependent recovery of fast *I*_Ca_. Only three recovery times (1, 128, 2048 ms) are shown for clarity. (I) Dots/error: Mean ± SEM of fast *I*_Ca_ tested at 0 mV with different recovery time on a log scale. Data were normalized to the maximum value obtained after 2048 ms recovery. Lines: Monophasic exponential association fits of time‐dependent recovery curve. (J) Half recovery time (*T*_0.5 recov_) of fast *I*_Ca_. Holm‐Sidak post hoc. RX, prenatal treatment.

Representative traces of voltage‐ and time‐dependent inactivation, and time‐dependent recovery from inactivation of fast *I*_Ca_ are in Figure 4A,E,H. PNA treatment shifted the voltage‐dependent inactivation curve of GnRH neurons to the right in both 3 week and adult groups (Figure 4B, Table 6). Half inactivation potential (*V*_0.5 inact_) was depolarized in PNA groups, suggesting resistance to voltage‐dependent inactivation (Figure 4C, Table 7). The voltage‐dependent inactivation slope factor was not different among groups (Figure 4D). For time‐dependent inactivation of the fast *I*_Ca_, GnRH neurons from adult mice exhibited a slower inactivation compared to cells from 3 week groups (Figure 4F, Table 6). Half inactivation time (*T*_0.5 inact_) was longer in cells from adult PNA than 3 week PNA mice (Figure 4G, Table 7). Neither age nor PNA treatment altered time‐dependent recovery of the fast *I*_Ca_ (Figure 4I,J, Tables 6, 7).

**TABLE 6:** Three‐way ANOVA/mixed effects analysis of fast component inactivation (Figure 4).

Voltage‐dependent inactivation	
Source of variation	Voltage‐dependent inactivation
Voltage	***F* (7, 323) = 540.3, *p*< .0001**
Age	*F* (1, 69) = 0.1328, *p* = .7167
Treatment	***F* (1, 69) = 6.196**, ***p* = .0152**
Voltage × age	*F* (7, 323) = 0.8191, *p* = .5719
Voltage × treatment	***F* (7, 323) = 2.673, *p* = .0106**
Age × treatment	*F* (1, 69) = 0.1502, *p* = .6996
Voltage × age × treatment	*F* (7, 323) = 0.2909, *p* = .9573

Time‐dependent inactivation and recovery		
Source of variation	Time‐dependent inactivation	Time‐dependent recovery
Time	***F* (7, 301) = 572.2**, ***p*< .0001**	***F* (11, 407) = 1125**, ***p*< .0001**
Age	***F* (1, 43) = 11.52**, ***p* = .0015**	*F* (1, 37) = 0.4679, *p* = .4982
Treatment	*F* (1, 43) = 0.3162, *p* = .5768	*F* (1, 37) = 2.367, *p* = .1324
Time × age	***F* (7, 301) = 3.061**, ***p* = .0040**	*F* (11, 407) = 0.4952, *p* = .9062
Time × treatment	*F* (7, 301) = 1.481, *p* = .1734	*F* (11, 407) = 0.8101, *p* = .6300
Age × treatment	*F* (1, 43) = 0.09984, *p* = .7536	*F* (1, 37) = 0.3475, *p* = .5591
Time × age × treatment	*F* (7, 301) = 0.5131, *p* = .8245	*F* (11, 407) = 1.191, *p* = .2909

*Note*: Bold indicates *p*< .05.

**TABLE 7:** Two‐way ANOVA of inactivation properties (Figure 4).

Property	Age	Treatment	Interaction
*V*_0.5 inact_	*F* (1, 68) = 0.2172, *p* = .6426	***F* (1, 68) = 5.993**, ***p* = .0169**	*F* (1, 68) = 0.005608, *p* = .9405
Voltage‐dependent inactivation slope factor	*F* (1, 66) = 0.1908, *p* = .6637	*F* (1, 66) = 0.3048, *p* = .5827	*F* (1, 66) = 0.01175, *p* = .9140
*T*_0.5 inact_	***F* (1, 44) = 11.63**, ***p* = .0014**	*F* (1, 44) = 0.3672, *p* = .5476	*F* (1, 44) = 0.1003, *p* = .7529
*T*_0.5 recov_	*F* (1, 37) = 0.9851, *p* = .3274	*F* (1, 37) = 1.565, *p* = .2188	*F* (1, 37) = 1.337, *p* = .2551

Holm‐Sidak analysis of significant two‐way ANOVA				
	3 week VEH versus 3 week PNA	Adult VEH versus adult PNA	3 week VEH versus adult VEH	3 week PNA versus adult PNA
*V*_0.5 inact_	*p* = .2964	*p* = .2964	*p* = .9116	*p* = .9116
*T*_0.5 inact_	*p* = .7366	*p* = .8486	*p* = .1223	***p* = .0320**

*Note*: Bold indicates *p*< .05.

### 3.5 GnRH neurons of 3 week PNA mice exhibit adult‐like firing response to current injections

**FIGURE 5:**
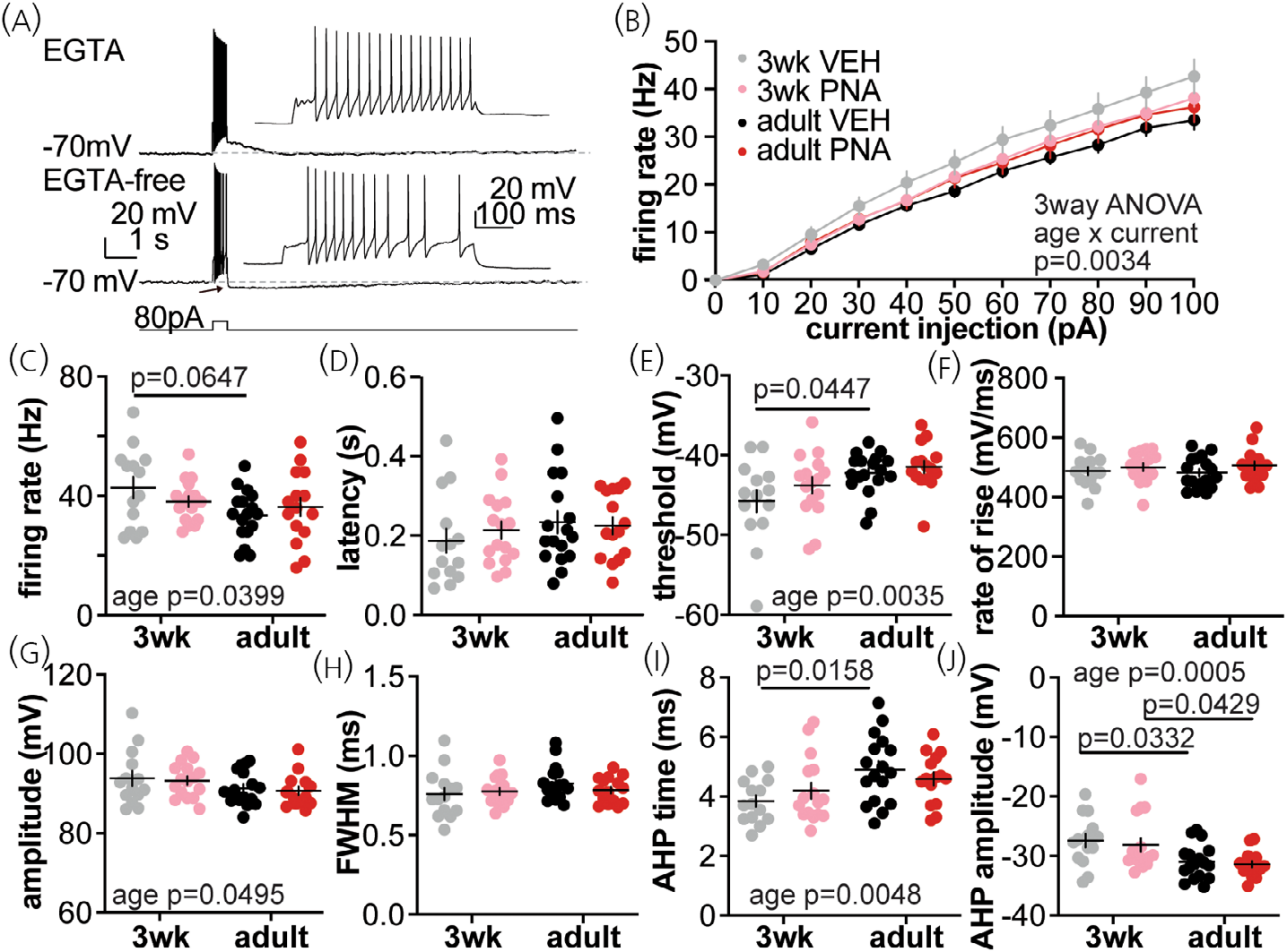
Intrinsic excitability of GnRH neurons in VEH and PNA mice during development. (A) Representative recordings of a GnRH neuron membrane potential in response to 80 pA current injection using EGTA‐containing (top) versus EGTA‐free pipette solution (bottom, used for the rest of the recordings, arrow shows post‐spike‐train hyperpolarization). Insets show individual action potentials. (B) Mean ± SEM firing rate during current injections from 10 to 100 pA with EGTA‐free solutions. (C) Firing rate during 100 pA current injection. (D)–(J), the first action potential latency (D), threshold (E), rise rate (F), amplitude relative to threshold (G), full‐width at half maximum (H), AHP time (I), AHP amplitude relative to threshold (J). Holm‐Sidak post hoc.

The excitability of GnRH neurons was quantified as the firing rate of action potentials (Hz) in response to 500 ms current injections during whole‐cell current‐clamp recordings. In pilot experiments, we used EGTA‐containing versus EGTA‐free pipette solution to assess the effects of exogenous Ca^2+^ buffering on GnRH neuron action potential firing and membrane potential trajectory after current injection. Fewer spikes were generated in response to current injections when GnRH neurons were recorded with EGTA‐free compared to those recorded using EGTA‐containing pipette solution (two‐way ANOVA current *F* (6, 42) = 38.82, *p*< .0001; EGTA F(1, 7) = 6.362, *p* = .0397; current × EGTA *F* (6, 42) *p*< .0001). A post‐spike‐train membrane hyperpolarization was observed when EGTA‐free solution was used (Figure 5A bottom, arrow), which was not observed in recordings using EGTA‐containing solution (Figure 5A top). We thus used EGTA‐free pipette solution in recordings to minimize effects of buffering on neuron excitability. GnRH neuron capacitance was higher in adult compared to 3 week‐old groups, while PNA treatment had no effect; *R*_in_, *R*_s_, and *I*_hold_ were not different among groups (Table 8). Three‐way ANOVA of firing rate in response to current injection steps revealed an effect of age on GnRH neurons excitability (Figure 5B, Table 9). Two‐way ANOVA of firing rate in response to 100 pA injection confirmed the effect of age on firing rate (Figure 5C, age *F* (1, 58) = 4.418, *p* = .0399). This developmental effect appears to be driven primarily by 3 week versus adult VEH as the post hoc test approached the level accepted for significance (*p* = .0647 Holm‐Sidak). In contrast, PNA 3 week‐old mice exhibit adult‐like firing response to current injection, and no age‐dependent effect was observed (*p* = .7050).

**TABLE 8:** Descriptive statistics and two‐way ANOVA parameters for passive properties and recording quality for GnRH neuron excitability recording (Figure 5).

Descriptive statistics				
Property	3 week VEH	3 week PNA	Adult VEH	Adult PNA
Series resistance (MΩ)	16.594 ± 0.770	16.427 ± 0.717	15.511 ± 1.062	14.833 ± 0.554
Input resistance (MΩ)	1280.424 ± 100.633	1098.038 ± 75.181	1192.588 ± 62.423	1222.051 ± 79.011
Capacitance (pF)	12.739 ± 0.689	13.202 ± 0.623	15.045 ± 0.568	14.371 ± 0.899
Holding current (pA)	−6.327 ± 2.300	−7.228 ± 2.179	−5.550 ± 1.295	−5.752 ± 1.956

Two‐way ANOVA			
	Age	Treatment	Interaction
Series resistance (MΩ)	*F* (1, 58) = 2.660, *p* = .1083	*F* (1, 58) = 0.2646, *p* = .6090	*F* (1, 58) = 0.09678, *p* = .7568
Input resistance (MΩ)	*F* (1, 58) = 0.05253, *p* = .8195	*F* (1, 58) = 0.9386, *p* = .3367	*F* (1, 58) = 1.801, *p* = .1848
Capacitance (pF)	***F* (1, 58) = 6.175**, ***p* = .0159**	*F* (1, 58) = 0.02271, *p* = .8807	*F* (1, 58) = 0.4195, *p* = .2669
Holding current (pA)	*F* (1, 58) = 0.5583, *p* = .4579	*F* (1, 58) = 0.2039, *p* = .6533	*F* (1, 58) = 0.0001689, *p* = .9897

Holm‐Sidak analysis of significant two‐way ANOVA				
	3 week VEH versus 3 week PNA	Adult VEH versus adult PNA	3 week VEH versus adult VEH	3 week PNA versus adult PNA
Capacitance (pF)	*p* = .7411	*p* = .7411	*p* = .0906	*p* = .5627

*Note*: Bold indicates *p*< .05.

**TABLE 9:** Three‐way ANOVA of GnRH neuron excitability (Figure 5).

Source of variation	
Current	***F* (10, 580) = 697.7**, ***p*< .0001**
Age	*F* (1, 58) = 3.424, *p* = .0693
Treatment	*F* (1, 58) = 0.1387, *p* = .7110
Current × age	***F* (10, 580) = 2.667**, ***p* = .0034**
Current × treatment	*F* (10, 580) = 0.1988, *p* = .9964
Age × treatment	*F* (1, 58) = 2.389, *p* = .1276
Current × age × treatment	*F* (10, 580) = 1.307, *p* = .2228

*Note*: Bold indicates *p*< .05.

The properties of the first action potential generated in EGTA‐free recordings were analyzed by two‐way ANOVA (Figure 5D–J, Table 10). Action potential threshold was hyperpolarized in GnRH neurons from 3 week versus adult VEH mice (Figure 5E), and the peak time of the AHP was earlier (Figure 5I). The amplitude of AHP relative to threshold was lower in cells from 3 week than those from adults in both VEH and PNA groups (Figure 5J). Action potential amplitude relative to threshold was higher in GnRH neurons from 3 week‐old mice compared with those from adults (Figure 5G). There was no difference in latency to fire in response to current injection, action potential rate of rise, or FWHM (Figure 5D,F,H).

**TABLE 10:** Two‐way ANOVA of first action potential properties (Figure 5).

Property	Age	Treatment	Interaction
Latency	*F* (1, 58) = 1.287, *p* = .2613	*F* (1, 58) = 0.1300, *p* = .7197	*F* (1, 58) = 0.5075, *p* = .4791
Threshold	***F* (1, 59) = 9.253**, ***p* = .0035**	*F* (1, 59) = 2.082, *p* = .1543	*F* (1, 59) = 0.4062, *p* = .5263
Rate of rise	F (1, 58) = 0.0003850, *p* = .9844	*F* (1, 58) = 2.020, *p* = .1606	*F* (1, 58) = 0.2035, *p* = .6536
AP amplitude	***F* (1, 58) = 4.026**, ***p* = .0495**	*F* (1, 58) = 0.2041, *p* = .6531	*F* (1, 58) = 1.866e‐006, *p* = .9989
FWHM	*F* (1, 58) = 1.864, *p* = .1774	*F* (1, 58) = 0.2212, *p* = .6399	*F* (1, 58) = 1.195, *p* = .2789
AHP time	***F* (1, 58) = 8.584**, ***p* = .0048**	*F* (1, 58) = 0.01039, *p* = .9192	*F* (1, 58) = 1.747, *p* = .1914
AHP amplitude	***F* (1, 58) = 13.78**, ***p* = .0005**	*F* (1, 58) = 0.3468, *p* = .5582	*F* (1, 58) = 0.02400, *p* = .8774

Holm‐Sidak analysis of significant two‐way ANOVA				
	3 week VEH versus 3 week PNA	Adult VEH versus adult PNA	3 week VEH versus adult VEH	3 week PNA versus adult PNA
Threshold	*p* = .2869	*p* = .5620	***p* = .0447**	*p* = .2606
Amplitude	*p* = .9361	*p* = .9361	*p* = .5039	*p* = .5039
AHP time	*p* = .5935	*p* = .5935	***p* = .0158**	*p* = .5935
AHP amplitude	*p* = .8453	*p* = .8453	***p* = .0332**	***p* = .0429**

*Note*: Bold indicates *p*<. 05.

### 3.6 Blocking small‐conductance Ca^2+^‐activated K^+^ (SK) current increases GnRH neuron firing rate except in adult PNA mice

**FIGURE 6:**
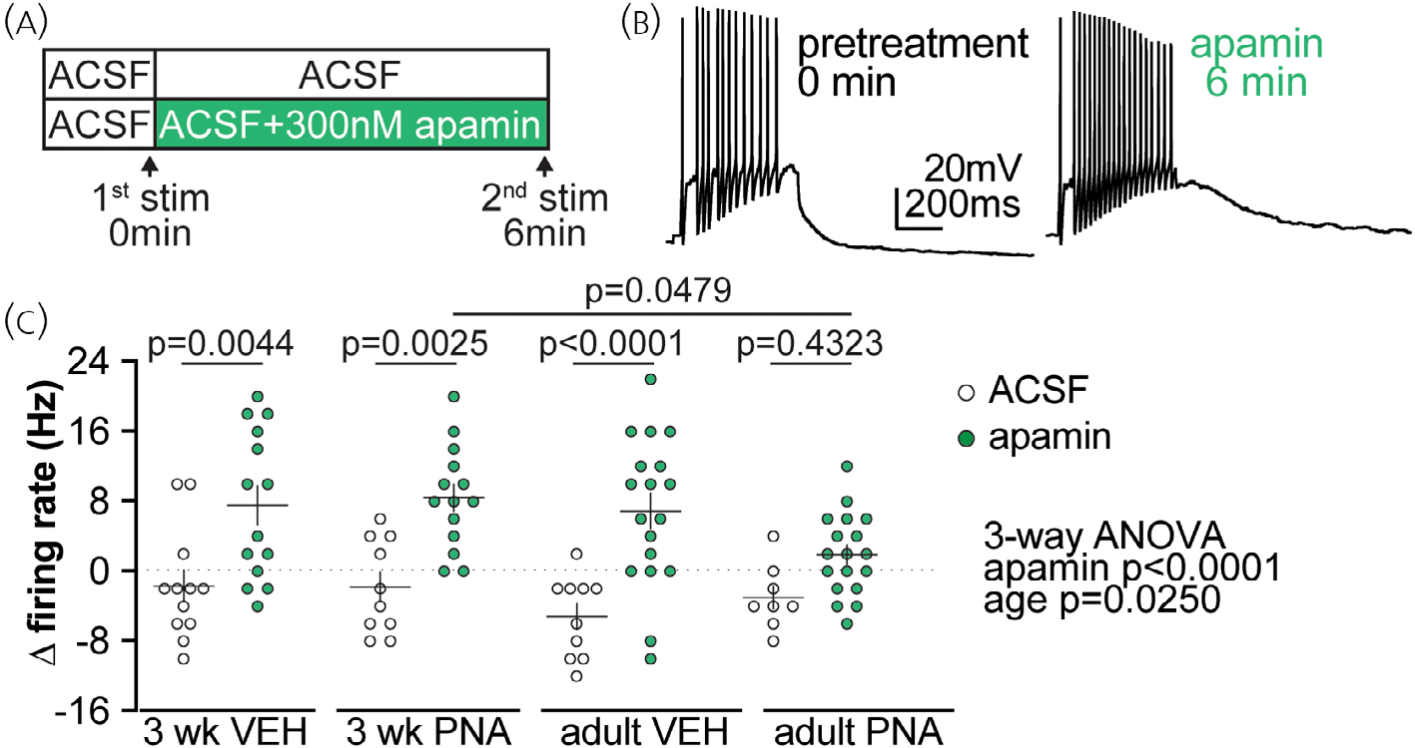
Effect of apamin perfusion on the firing rate of GnRH neurons from 3 week‐old and adult VEH and PNA mice. (A) Experimental timeline. (B) Representative action potential firing of a GnRH neuron from a 3 week VEH mouse in response to 80 pA current injection at 0 min pretreatment and after 6 min of apamin perfusion. (C) Change in firing rate for each cell from pretreatment to 6 min after continued perfusion of ACSF (white) or change to apamin (green). Holm‐Sidak post hoc.

During repeated firing, calcium influx and [Ca^2+^]_in_ could activate SK channels, leading to a hyperpolarizing SK current. We tested the role of the SK current on GnRH neuron intrinsic excitability by blocking it with apamin (Figure 6A,B). As in the other experiments, GnRH neuron capacitance was higher in adult compared to 3 week‐old groups, while PNA treatment had no effect; *R*_in_, *R*_s_, and *I*_hold_ were not different among groups (Table 11, two‐way ANOVA). GnRH neuron firing response to a 500 ms, 80 pA current injection was recorded under basal conditions (0 min) and 6 min after the onset of apamin or ACSF control perfusion, and the change of firing rate calculated (Figure 6C). Three‐way ANOVA revealed both apamin and age affected the change in firing rate. Most ACSF control cells exhibited a mildly negative change in firing rate (Figure 6C), and there was no difference in the change in firing rate among ACSF control groups. In contrast, cells treated with apamin to block SK current largely exhibited an increase in firing rate, with the exception of cells from adult PNA mice (Figure 6C, Table 12). These results indicated a blunted response to apamin in GnRH neurons from adult PNA mice.

**TABLE 11:** Descriptive statistics and two‐way ANOVA parameters for passive properties and recording quality for GnRH neuron excitability recording with vehicle or apamin perfusion (Figures 6, 7, 8).

Descriptive statistics				
Property	3 week VEH	3 week PNA	Adult VEH	Adult PNA
Series resistance (MΩ)	17.401 ± 0.646	16.656 ± 0.884	16.736 ± 0.655	18.351 ± 0.735
Input resistance (MΩ)	1510.862 ± 94.748	1519.596 ± 91.429	1408.678 ± 93.477	1352.456 ± 79.550
Capacitance (pF)	12.390 ± 0.353	13.194 ± 0.339	15.921 ± 0.418	15.112 ± 0.537
Holding current (pA)	−5.731 ± 1.300	−2.695 ± 1.093	−2.061 ± 1.286	−1.785 ± 1.606

Two‐way ANOVA			
	Age	Treatment	Interaction
Series resistance (MΩ)	*F* (1, 100) = 0.4987, *p* = .4817	*F* (1, 100) = 0.3560, *p* = .5521	*F* (1, 100) = 2.617, *p* = .1089
Input resistance (MΩ)	*F* (1, 100) = 2.221, *p* = .1393	*F* (1, 100) = 0.06904, *p* = .7933	*F* (1, 100) = 0.1292, *p* = .7201
Capacitance (pF)	*F* (1, 100) = 36.99, ***p*< .0001**	*F* (1, 100) = 0.01855, *p* = .8919	*F* (1, 100) = 3.631, *p* = .0596
Holding current (pA)	*F* (1, 100) = 2.901, *p* = .0916	*F* (1, 100) = 1.516, *p* = .2211	*F* (1, 100) = 1.054, *p* = .3071

Holm‐Sidak analysis of significant two‐way ANOVA				
	3 week VEH versus 3 week PNA	Adult VEH versus adult PNA	3 week VEH versus adult VEH	3 week PNA versus adult PNA
Capacitance (pF)	*p* = .3134	*p* = .3134	***p*< .0001**	***p* = .0065**

*Note*: Bold indicates *p*< .05.

**TABLE 12:** Three‐way ANOVA of the effects of apamin (Figure 6).

Source of variation	
Apamin	*F* (1, 96) = 48.85, ***p*< .0001**
Age	*F* (1, 96) = 5.184, ***p* = .0250**
Treatment	*F* (1, 96) = 0.1563, *p* = .6934
Apamin × age	*F* (1, 96) = 0.2247, *p* = .6366
Apamin × treatment	*F* (1, 96) = 1.399, *p* = .2399
Age × treatment	*F* (1, 96) = 0.4504, *p* = .5038
Apamin × age × treatment	*F* (1, 96) = 2.433, *p* = .1221

Holm‐Sidak analysis of significant three‐way ANOVA	
Control: 3 week VEH versus 3 week PNA	*p* = .9914
Control: adult VEH versus adult PNA	*p* = .9597
Control: 3 week VEH versus adult VEH	*p* = .7456
Control: 3 week PNA versus adult PNA	*p* = .9914
3 week VEH: control versus apamin	***p* = .0044**
3 week PNA: control versus apamin	***p* = .0025**
Adult VEH: control versus apamin	***p*< .0001**
Adult PNA: control versus apamin	*p* = .4323
Apamin: 3 week VEH versus 3 week PNA	*p* = .9914
Apamin: adult VEH versus adult PNA	*p* = .1643
Apamin: 3 week VEH versus adult VEH	*p* = .9914
Apamin: 3 week PNA versus adult PNA	***p* = .0479**

*Note*: Bold indicates *p*< .05.

### 3.7 Components of post‐spike‐train membrane potential changes are altered in GnRH neurons from PNA mice

**FIGURE 7:**
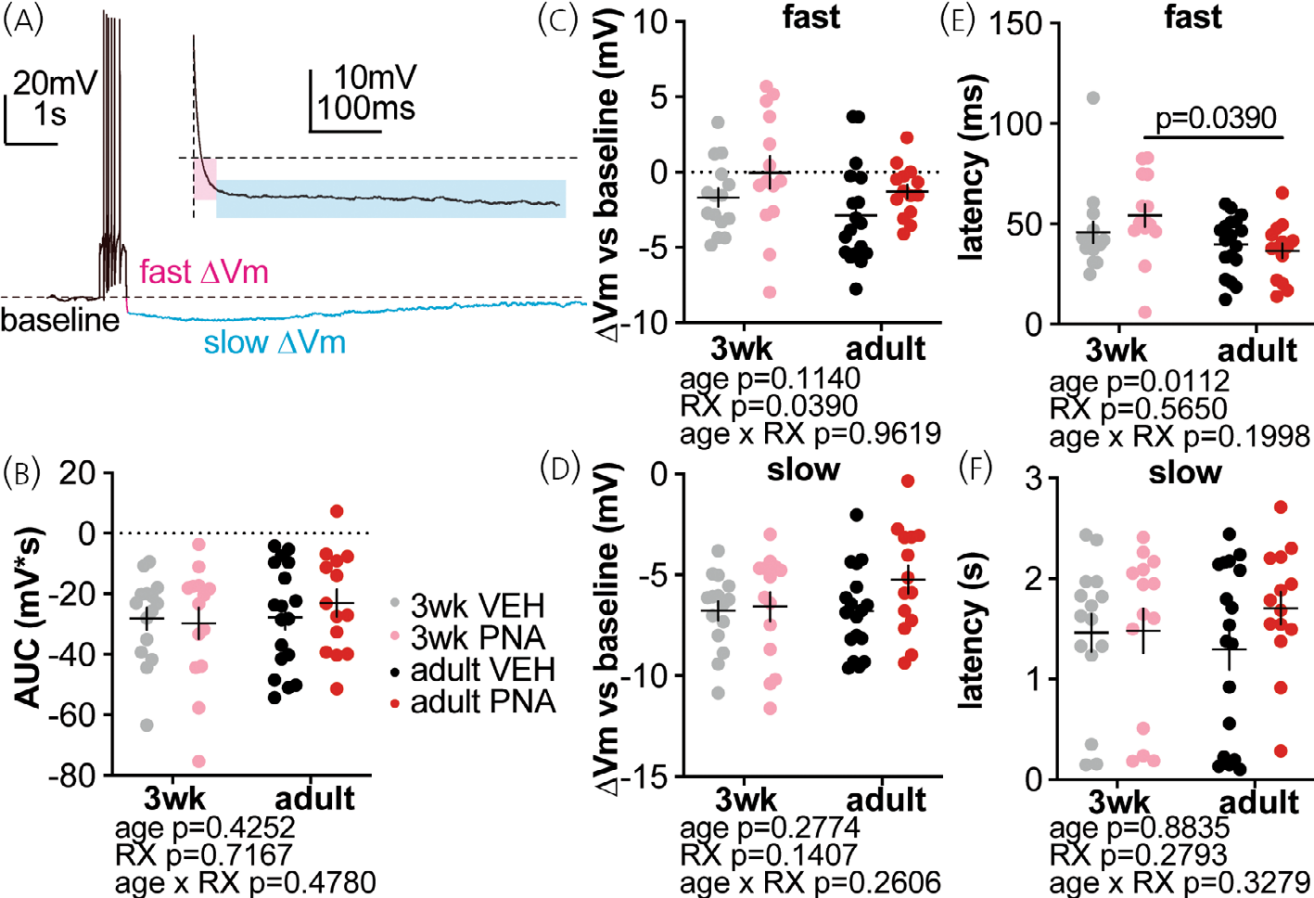
Change in post‐spike‐train membrane potential (Δ*V*_m_) relative to baseline potential under basal conditions. (A) Representative cell from an adult VEH mouse in response to 80 pA current injection. Horizontal dashed line: Pre‐stimulus baseline; magenta to cyan color change marks repolarization rate falling below 50 mV/s. Insert: Expanded trace showing fast Δ*V*_m_ (magenta box) and slow ∆*V*_m_ (cyan box); vertical dashed line: End of current injection. (B) Post‐spike‐train Δ*V*_m_ area under the curve (AUC) for the 12.5 s analysis period. (C), (E) Post‐spike‐train fast Δ*V*_m_ peak value (C) and latency (E). (D), (F) Post‐spike‐train slow Δ*V*_m_ peak value (D) and latency (F). RX, prenatal treatment. Holm‐Sidak post hoc.

At the end of the 80 pA current injection under basal conditions (before apamin), the trajectory of the membrane potential change exhibited two phases. Immediately after termination of the current injection, most cells rapidly repolarized, with 46 of 61 cells across the four groups showing fast membrane hyperpolarization (fast post‐spike‐train ∆*V*_m_ relative to baseline potential, magenta Figure 7A). All cells then exhibited a more prolonged, slow hyperpolarization, which gradually returned to baseline potential over several seconds (slow post‐spike‐train ∆*V*_m_ relative to baseline potential, cyan Figure 7A). The AUC of ∆*V*_m_ relative to baseline potential was not different among groups during the 12.5 s post‐stimulus period (Figure 7B, Table 13). To quantify the fast versus slow post‐spike‐train ∆*V*_m_, the inflection point when the repolarization rate after current injection termination fell below 50 mV/s was used, with the period before being designated fast and that after slow (Figure 7A). The fast Δ*V*_m_ relative to baseline potential was shifted toward zero or positive values in cells from PNA mice (Figure 7C, Table 13). The latency to reach the inflection point was longer in GnRH neurons from 3 week PNA than adult PNA mice, suggesting a prolonged post‐spike‐train repolarization/hyperpolarization phase in younger PNA animals (Figure 7E, Table 13). The peak value and latency time of post‐spike‐train slow Δ*V*_m_ were not different among groups (Figure 7D,F).

**TABLE 13:** Two‐way ANOVA of post‐spike train change in membrane potential under basal conditions (Figure 7).

Property	Age	Treatment	Interaction
Δ*V*_m_ AUC	*F* (1, 57) = 0.6452, *p* = .4252	*F* (1, 57) = 0.1330, *p* = .7167	*F* (1, 57) = 0.5149, *p* = .4780
Fast Δ*V*_m_ versus baseline	*F* (1, 57) = 2.576, *p* = .1140	***F* (1, 57) = 4.462**, ***p* = .0390**	*F* (1, 57) = 0.002304, *p* = .9619
Fast Δ*V*_m_ latency	***F* (1, 57) = 6.870**, ***p* = .0112**	*F* (1, 57) = 0.3350, *p* = .5650	*F* (1, 57) = 1.683, *p* = .1998
Slow Δ*V*_m_ versus baseline	*F* (1, 57) = 1.203, *p* = .2774	*F* (1, 57) = 2.232, *p* = .1407	*F* (1, 57) = 1.291, *p* = .2606
Slow Δ*V*_m_ latency	*F* (1, 57) = 0.02167, *p* = .8835	*F* (1, 57) = 1.193, *p* = .2793	*F* (1, 57) = 0.9738, *p* = .3279

Holm‐Sidak analysis of significant two‐way ANOVA				
	3 week VEH versus 3 week PNA	Adult VEH versus adult PNA	3 week VEH versus adult VEH	3 week PNA versus adult PNA
Fast Δ*V*_m_ versus baseline	*p* = .4530	*p* = .4530	*p* = .4530	*p* = .4530
Fast Δ*V*_m_ latency	*p* = .4860	*p* = .6058	*p* = .5562	***p* = .0390**

*Note*: Bold indicates *p*< .05.

**FIGURE 8:**
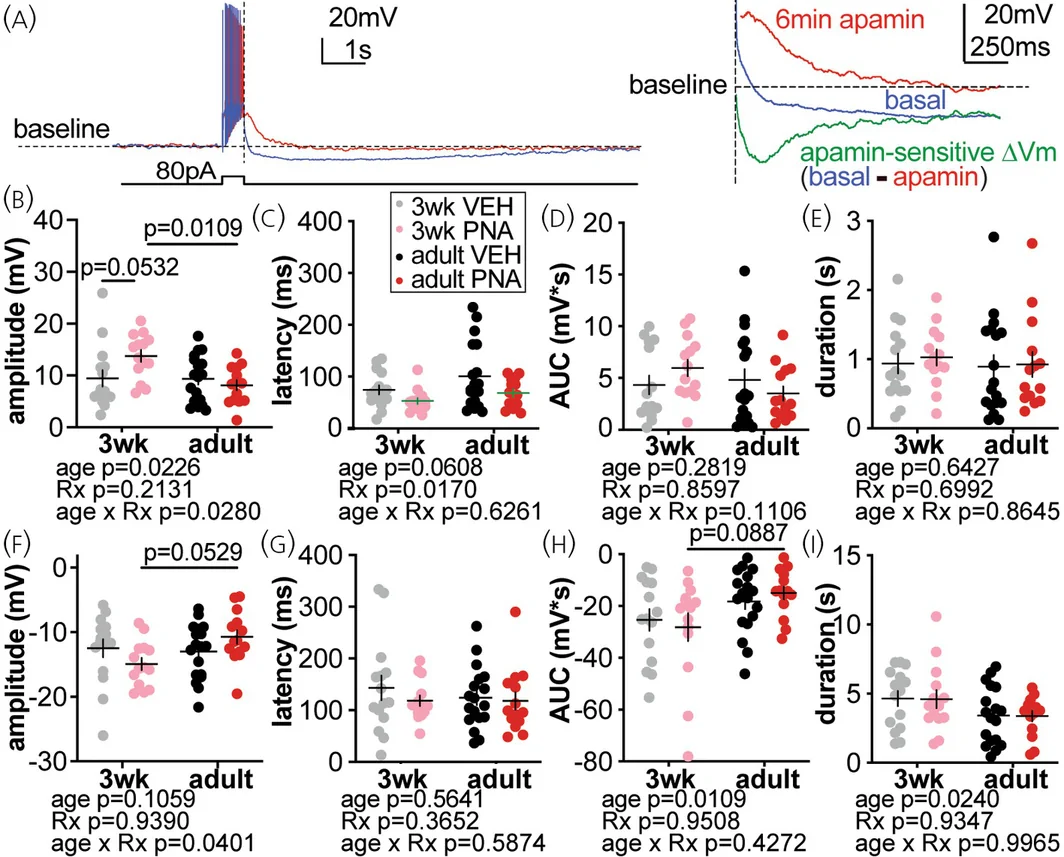
The post‐spike‐train depolarization unmasked by apamin, and apamin‐sensitive SK‐mediated hyperpolarization. (A) Left: Representative membrane potentials from the same GnRH neuron in response to 80 pA current injection under basal conditions (blue) and after 6 min of apamin treatment (red). Right: Enlargement of the figure on the left showing post‐spike‐train membrane potentials under basal conditions (blue), after apamin treatment (red) and the SK‐mediated hyperpolarization (green) obtained by subtracting the red trace from the blue trace. Horizontal dashed line: Baseline potential; vertical dashed line: End of current injection. (B)–(E), post‐spike‐train depolarization peak value (B), latency (C), AUC at 10% maximum (D), and duration at 10% maximum (E). (F)–(I), SK‐mediated apamin‐sensitive hyperpolarization peak value (F), latency (G), AUC at 10% maximum (H), and duration at 10% maximum (I). RX, prenatal treatment. Holm‐Sidak post hoc.

The 6‐min apamin treatment changed the post‐spike‐train membrane potential trajectory in all groups, unmasking a fast depolarization that peaked within 250 ms of the end of current injection (Figure 8A, red trace, Figure 8C). The amplitude of post‐spike‐train depolarization was influenced by age and an interaction between age and PNA treatment (Figure 8B, Table 14). Specifically, GnRH neurons from 3 week PNA mice had a higher post‐spike‐train depolarization amplitude, which decreased in adulthood to values observed in VEH mice. The latency to peak of the depolarization was shortened in PNA groups (Figure 8C, Table 14). To minimize the influence of noise and prolonged tails when quantifying the AUC and duration of the post‐spike‐train depolarization, the onset and termination were defined as the point when the potential crossed 10% of the depolarization peak value (AUC at 10% maximum, duration at 10% maximum). There was no difference in post‐spike‐train depolarization AUC or duration at 10% maximum among groups (Figure 8D,E).

**TABLE 14:** Two‐way ANOVA of the effects of apamin on post‐spike train change in membrane potential (Figure 8).

Post‐spike‐train depolarization			
Property	Age	Treatment	Interaction
Amplitude	***F* (1, 57) = 5.495**, ***p* = .0226**	*F* (1, 57) = 1.586, *p* = .2131	***F* (1, 57) = 5.087**, ***p* = .0280**
Latency	*F* (1, 57) = 3.658, *p* = .0608	***F* (1, 57) = 6.045**, ***p* = .0170**	*F* (1, 57) = 0.2400, *p* = .6261
AUC	*F* (1, 57) = 1.180, *p* = .2819	*F* (1, 57) = 0.03154, *p* = .8597	*F* (1, 57) = 2.627, *p* = .1106
Duration	*F* (1, 57) = 0.2176, *p* = .6427	*F* (1, 57) = 0.1508, *p* = .6992	*F* (1, 57) = 0.02939, *p* = .8645

Holm‐Sidak analysis of significant two‐way ANOVA				
	3 week VEH versus 3 week PNA	Adult VEH versus adult PNA	3 week VEH versus adult VEH	3 week PNA versus adult PNA
Amplitude	*p* = .0532	*p* = .7240	*p* = .9482	***p* = .0109**
Latency	*p* = .3243	*p* = .1418	*p* = .2270	*p* = .3367

Apamin‐sensitive (SK‐mediated) hyperpolarization			
Property	Age	Treatment	Interaction
Amplitude	*F* (1, 57) = 2.700, *p* = .1059	*F* (1, 57) = 0.005912, *p* = .9390	**F (1, 57) = 4.413**, ***p* = .0401**
Latency	*F* (1, 57) = 0.3365, *p* = .5641	*F* (1, 57) = 0.8331, *p* = .3652	F (1, 57) = 0.2977, *p* = .5874
AUC	***F* (1, 57) = 6.929**, ***p* = .0109**	*F* (1, 57) = 0.003836, *p* = .9508	F (1, 57) = 0.6396, *p* = .4272
Duration	***F* (1, 57) = 5.379**, ***p* = .0240**	*F* (1, 57) = 0.006762, *p* = .9347	F (1, 57) = 1.945e‐005, *p* = .9965

Holm‐Sidak analysis of significant two‐way ANOVA				
	3 week VEH versus 3 week PNA	Adult VEH versus adult PNA	3 week VEH versus adult VEH	3 week PNA versus adult PNA
Amplitude	*p* = .3571	*p* = .3571	*p* = .7373	*p* = .0529
AUC	*p* = .7848	*p* = .7848	*p* = .4538	*p* = .0887
Duration	*p* = .9975	*p* = .9975	*p* = .3254	*p* = .3254

*Note*: Bold indicates *p*< .05.

The change in post‐spike‐train membrane potential following apamin perfusion indicates that the apamin‐sensitive SK current shapes the membrane potential after a train of action potentials, counteracting depolarization and facilitating post‐spike‐train repolarization/hyperpolarization. To assess the net effect of apamin‐sensitive SK current on post‐spike‐train membrane potential, we subtracted the membrane potential trace recorded 6 min with apamin perfusion from that recorded in the basal state before treatment. The subtraction revealed a SK‐mediated hyperpolarization that peaked within 350 ms of the end of current injection (Figure 8A, green trace, Figure 8G). The amplitude of SK‐mediated hyperpolarization was influenced by an interaction between age and PNA treatment, with a strong trend of greater SK‐mediated hyperpolarization in GnRH neurons from 3 week PNA mice that declined in adulthood (Figure 8F, Table 14). AUC of the SK‐mediated hyperpolarization behaved in a similar manner (Figure 8H). There was no difference in the latency to the SK‐mediated hyperpolarization peak (Figure 8G). Compared with 3 week groups, GnRH neurons from adult groups had shorter duration of SK‐mediated hyperpolarization regardless of PNA treatment (Figure 8I).

## 4 DISCUSSION

Precise control of the frequency of GnRH pulses is required to drive the female reproductive cycle. In women with hyperandrogenemic PMOS, typical frequency changes in LH pulses, which reflect GnRH pulses, are replaced by persistent high frequency pulses. Similarly, LH‐pulse frequency is elevated in PNA mice, and these mice lack the developmental changes in spontaneous GnRH neuron firing activity observed in VEH mice. Here we examined age and PNA‐induced changes in voltage‐gated Ca^2+^ currents, which are critical to excitation‐secretion coupling,^30^, ^36^ and firing dynamics.^31^, ^37^, ^38^ Calcium currents were altered by PNA treatment and by development, indicating they may contribute to the phenotypes in PNA mice and possibly women with PMOS as well as to normal puberty.

The present results support prior studies of GnRH neuron *I*_Ca_ that report the total current is comprised of medium‐slow and fast inactivating components. The medium‐slow *I*_Ca_ displays slow voltage‐dependent inactivation characteristic of L‐type Ca^2+^ channels,^39^, ^40^, ^41^ whereas the fast *I*_Ca_ component is likely mediated by N, P/Q, and R Ca^2+^ channels, with a minimal T‐type component. Our data extend prior studies by demonstrating that GnRH neurons from PNA mice exhibited increased *I*_Ca_ density compared with VEH groups at both peripubertal and adult ages for total *I*_Ca_, as well as both the medium‐slow and fast subcomponents. The among‐group differences were evident at depolarized membrane potentials consistent with activation of high‐voltage‐activated calcium channels. In contrast, no differences were observed at more hyperpolarized potentials that would tend to activate low‐voltage‐activated T‐type currents. Minimal current activation at those potentials is consistent with prior reports^42^, ^43^, ^44^ and with reports that Ca_V_3 subunits encoding T‐type channels are expressed only in a subset of GnRH neurons.^45^ Altered T‐type *I*_Ca_ is thus not likely a major cause of development or PNA‐induced changes in GnRH neuron activity.^25^

*I*_Ca_ properties depended on both PNA treatment and age. GnRH neurons exhibited a developmental depolarization in voltage‐dependent activation of total Ca^2+^, likely attributable to the medium‐slow component. The more hyperpolarized activation of Ca^2+^ channels in GnRH neurons from 3 week mice may help sustain and facilitate membrane depolarization near action potential threshold^46^, ^47^ and could thus in part explain increased spontaneous action potential firing in the 3 week group, particularly within VEH mice.^25^ The half‐inactivation time of the fast *I*_Ca_ was longer in adult mice and this developmental change was more prominent in PNA mice. GnRH neurons from PNA mice had a depolarized half‐inactivation potential compared with VEH groups regardless of age. Along with increased *I*_Ca_ density, these changes in *I*_Ca_ inactivation properties in PNA mice may increase Ca^2+^ entry during excitation‐secretion coupling to facilitate neuropeptide release, potentially favoring higher GnRH pulse amplitude; this could also contribute to the pubertal transition. Of interest in this regard, LH‐pulse amplitude in women with PMOS is elevated compared to either early or mid‐follicular phase of control women.^48^ Alterations in mRNA expression levels of Ca^2+^ channel subtypes, and/or posttranslational modifications of Ca^2+^ channel *α* and/or *β* subunits could influence these properties.

Steroid milieu changes with both development and PNA and could underlie some of the observed shifts in *I*_Ca_ properties. More L‐type but less N‐type mediated currents were reported in 4–10‐day old mice compared with adults.^43^ In vivo estradiol treatment of adult ovariectomized mice at a level sufficient to induce a preovulatory GnRH/LH surge increases the expression of Ca_V_1.3, Ca_V_2.2, and Ca_V_2.3 channels in GnRH neurons, but had no effect on Ca_V_1.2.^49^ Ovariectomized female mice treated with estradiol leading to diurnal changes in GnRH neuron firing rate also exhibit diurnal changes in high‐voltage activated *I*_Ca_ amplitude with alterations in L‐and N‐type subtypes.^42^ Removing steroid negative feedback by castration increases Ca^2+^ currents in GnRH neurons from both male^30^ and female mice.^42^ Perhaps most relevant, treatment of ovariectomized mice with DHT increased *I*_Ca_ ^50^ and PNA mice have elevated testosterone levels.^22^, ^23^ Shifts in steroid milieu, particularly androgens, could thus mediate observed changes in *I*_*Ca*_.

GnRH neuron excitability was examined to assess functional changes in GnRH neuron output that the observed changes in *I*_Ca_ may contribute to, using an EGTA‐free pipette solution to minimize buffering. Firing rate in response to current injection steps revealed an effect of age on GnRH neuron excitability that was driven primarily by higher firing rate in 3 week VEH group; this developmental change was not evident in cells from PNA mice. These excitability changes resemble age‐ and PNA‐related changes in extracellularly recorded spontaneous firing activity of GnRH neurons.^25^ In studies using EGTA‐containing pipette solution, GnRH neurons from VEH and PNA mice had comparable excitability that increased with age.^29^ Differences with and without buffering suggested *I*_Ca_ and [Ca^2+^]_in_ play roles in GnRH neuron excitability in VEH and PNA mice during development. In the present work, threshold of the first action potential was hyperpolarized in cells from 3 week VEH mice compared with those from adults, consistent with the firing rate changes observed. Age also altered the afterhyperpolarization following this spike, with increased amplitude and a later peak time observed in adults. Since similar age‐dependent changes in the AHP were observed in studies using EGTA‐containing pipette solution,^29^ Na^+^ (threshold) and K^+^ currents (AHP)^28^ likely mediate these changes in first‐action potential properties.

During an action potential burst, increased [Ca^2+^]_in_ could open Ca^2+^‐activated K^+^ channels, a mechanism that may reduce GnRH neuron excitability by hyperpolarizing post‐spike membrane potential, thereby increasing intraspike intervals and terminating burst firing. In current‐clamp recordings under basal conditions, the post‐spike‐train fast Δ*V*_m_, which peaks within 150 ms after the termination of current injection, was shifted toward zero or became depolarizing in GnRH neurons from PNA mice regardless of age. This reduction in hyperpolarization may provide a transient window favoring another spike, thus prolonging a burst, and/or changing the availability of voltage‐dependent channels. Blockade of SK channels with apamin markedly reduced the post‐spike‐train membrane hyperpolarization, confirming the role for the rapidly activating apamin‐sensitive SK current.^31^ Interestingly, apamin increased GnRH neuron firing rate in response to current injection in all groups except PNA adults, suggesting a limited effect of SK channels in restraining firing in this group. GnRH neurons in PNA adult mice would therefore be more likely to have burst firing with prolonged duration,^51^ which may enhance the GnRH release efficiency and contribute to higher GnRH pulse amplitude.

Blocking SK channels with apamin unmasked a post‐spike‐train depolarization, termed an afterdepolarization potential,^52^ indicating the post‐spike‐train membrane potential is determined by a dynamic competition between depolarizing and hyperpolarizing currents, including SK. Afterdepolarizations are mediated by a TTX‐sensitive sodium current in GnRH neurons^52^ and increased firing is observed during this period. Interestingly, GnRH neurons from 3 week PNA mice exhibited the largest post‐spike‐train depolarization that is counterbalanced by a stronger SK‐mediated hyperpolarization. The stronger SK‐mediated hyperpolarization constrains firing, consistent with the observation that its blockade by apamin increased firing rate in the 3 week PNA group. This may help explain the lower firing rate of GnRH neurons from 3 week PNA versus 3 week VEH mice observed in published extracellular^25^ and the present current‐clamp recordings. The stronger SK‐mediated hyperpolarization may be an intrinsic homeostatic compensatory mechanism to counteract the increased excitatory GABAergic drive these cells receive at this age.^27^ In adulthood, the post‐spike‐train depolarization in GnRH neurons from PNA mice decreased to levels comparable to VEH mice, and this is accompanied by decreased SK‐mediated hyperpolarization. Consistent with this finding, the blockade of SK channels by apamin had no significant effect on GnRH neuron firing rate in the adult PNA group. In contrast, GnRH neurons from VEH mice had stable SK‐mediated hyperpolarization during development. GnRH neurons from adult PNA mice may thus have a reduced capacity to constrain burst firing with this mechanism. Thus, any compensation it provides for increased excitatory GABAergic drive, which persists into adulthood in PNA mice, would be lost. In addition to GABAergic inputs, blunted SK‐mediated hyperpolarization may increase the sensitivity of GnRH neurons to kisspeptin and/or neurokinin B released from the arcuate kisspeptin, neurokinin B, and dynorphin‐secreting network, the putative GnRH pulse generator.^53^ Although PNA treatment does not alter the spontaneous firing activity of those cells,^54^ increased neuropeptide sensitivity of GnRH neurons from adult PNA mice may change the peptide input needed to trigger burst firing, thereby increasing GnRH pulse frequency. It should be noted that the diminished SK‐mediated hyperpolarization in adult PNA GnRH neurons appeared despite increased *I*_Ca_, suggesting reduced coupling of increased *I*_Ca_ to the opening of SK channels.

Together these findings demonstrate that *I*_Ca_ in GnRH neurons is modified both by developmental stage and PNA treatment. These changes may contribute to the differential pubertal transition in VEH and PNA mice, and enhance Ca^2+^ entry in PNA mice during excitation‐secretion coupling, thereby increasing GnRH pulse amplitude. The transitory compensation mechanisms involving calcium‐activated potassium currents for excitatory drive appeared to be lost in adult PNA mice, potentially contributing to increased GnRH burst firing activity and, consequently, higher GnRH pulse amplitude and frequency. The study provides insights into mechanisms underlying GnRH hyperactivity in PNA mice, and sheds light on potential targets for PMOS treatment. Future studies of the role of *I*_Ca_ in GnRH neuron firing dynamics can be approached with dynamic clamp, whereas its role in excitation‐secretion coupling can be examined with real‐time measures of GnRH release with fast‐scan cyclic voltammetry.

## AUTHOR CONTRIBUTIONS

**Xi Chen:** Conceptualization; methodology; data curation; formal analysis; investigation; writing – original draft; writing – review and editing. **Suzanne M. Moenter:** Conceptualization; data curation; formal analysis; investigation; supervision; funding acquisition; writing – original draft; writing – review and editing; project administration. **R. Anthony DeFazio:** Conceptualization; formal analysis; writing – review and editing. **Jennifer Jaime:** Conceptualization; investigation; writing – review and editing.

## CONFLICT OF INTEREST STATEMENT

The authors declare no competing financial interests.

## Data Availability

The data that support the findings of this study are available from the corresponding author upon reasonable request.
